# A charge polarization model for the metal-specific activity of superoxide dismutases†
†Electronic supplementary information (ESI) available. See DOI: 10.1039/c7cp06829h


**DOI:** 10.1039/c7cp06829h

**Published:** 2017-12-18

**Authors:** Anna Barwinska-Sendra, Arnaud Baslé, Kevin J. Waldron, Sun Un

**Affiliations:** a Institute for Cell and Molecular Biosciences , Newcastle University , Newcastle upon Tyne , NE2 4HH , UK . Email: kevin.waldron@newcastle.ac.uk; b Department of Biochemistry , Biophysics and Structural Biology , Institute for Integrative Biology of the Cell (I2BC) , Université Paris-Saclay , CEA , CNRS UMR 9198 , CEA-Saclay , Gif-sur-Yvette , F-91198 , France . Email: sun.un@cea.fr

## Abstract

The pathogenicity of *Staphylococcus aureus* is enhanced by having two superoxide dismutases (SODs): a Mn-specific SOD and another that can use either Mn or Fe. Using 94 GHz electron-nuclear double resonance (ENDOR) and electron double resonance detected (ELDOR)-NMR we show that, despite their different metal-specificities, their structural and electronic similarities extend down to their active-site ^1^H– and ^14^N–Mn(ii) hyperfine interactions. However these interactions, and hence the positions of these nuclei, are different in the inactive Mn-reconstituted *Escherichia coli* Fe-specific SOD. Density functional theory modelling attributes this to a different angular position of the *E. coli* H171 ligand. This likely disrupts the Mn–H171–E170′ triad causing a shift in charge and in metal redox potential, leading to the loss of activity. This is supported by the correlated differences in the Mn(ii) zero-field interactions of the three SOD types and suggests that the triad is important for determining metal specific activity.

## Abstract 2

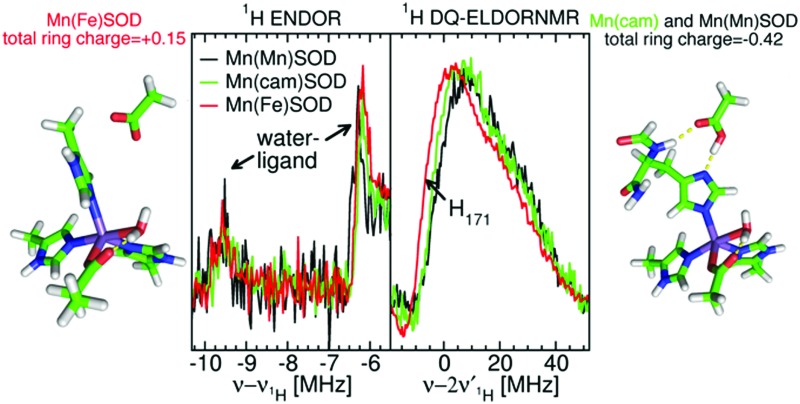
EPR shows that the angular position of the histidine-171 ligand likely plays an important role in metal-selective activities of Mn/FeSODs.

## Introduction

How organisms control the reactivities of essential metal ions, which are fundamental to the function of the approximately one-third of proteins that require a metal cofactor, is crucially important to their survival. For example, *Staphylococcus aureus* has a constitutive Mn-specific superoxide dismutase (herein MnSOD), which is catalytically inactive when loaded with Fe, and a second one (herein camSOD)^1^ that is stress-induced and cambialistic—that is it can use either Mn or Fe. This cofactor promiscuity enhances the pathogenicity of *S. aureus* by enabling it to survive the oxidative burst of the innate immune system while experiencing host-imposed Mn starvation.

**Fig. 1:**
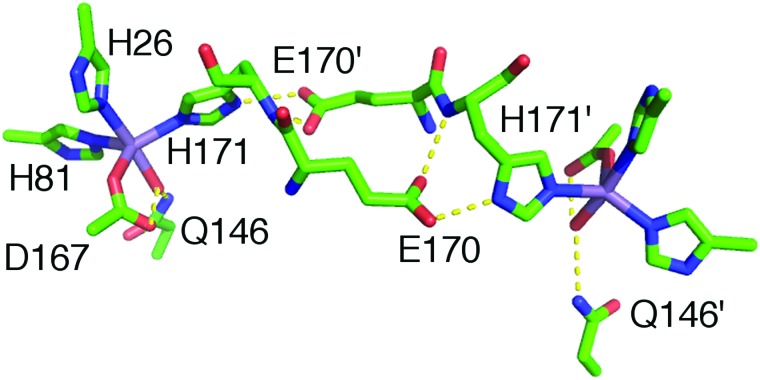
The structure of the interconnected metal sites of the two subunits of Mn/FeSODs, with carbon atoms in green, oxygen atoms in red, nitrogen atoms in blue, and the metal ion in purple. The residue numbering here, and in the text, is based on the familiar *E. coli* MnSOD (see the ESI†).

Metalloproteins can precisely control the chemistry of their metal cofactors, and the SODs are a remarkable example of this phenomenon. Mn-, cam- and Fe-specific SODs (herein FeSOD) can all bind both metals. The active sites of these SODs, composed of a metal ion and its five ligands (three histidines, an aspartate, and a water molecule, (Fig. 1)) in a distorted trigonal bipyramidal arrangement, are virtually identical to within the average 2.0 Å resolution found for the published MnSOD and Mn-loaded camSOD crystallographic structures (see the ESI†). In spite of this, Mn- and FeSODs display highly metal-specific activity. This behavior is thought to be conferred by protein redox tuning of the metal,^2^,^3^ although there are few structural clues as to how this is accomplished.

Unlike that of the Fe(iii)/Fe(ii) couple (0.77 V), the standard electrode potential (*E*^0^) of the aqueous complexes of Mn(iii)/Mn(ii) (1.51 V) is outside of the range required for catalytic SOD activity (between –0.16 and +0.89 V).^4^ Hence, the protein environments of MnSOD and FeSOD modify their respective cofactor's *E*^0^ in a differential manner, and achieve this feat while having active site structures that are nearly identical, extending well beyond the primary ligand sphere. Since stabilization of the water ligand over an OH^–^ will increase *E*^0^, favoring the M(ii) ion with respect to M(iii), it is likely that D167 and Q146, to which the water respectively donates and accepts a hydrogen-bond (Fig. 1), play a role in the metal redox tuning. The mutation of Q146 to glutamate in *E. coli* FeSOD reverses the hydrogen bonding direction to the water ligand and has been shown to increase *E*^0^ by more than 0.66 V.^5^ A similar range is observed for simple isostructural [M(4′-*R*-terpyridine)_2_]^2+^ complexes of Mn and Fe whose metal–ligand bond-lengths vary by less than 0.07 Å. Their *E*^0^ values are controlled by the electron-donating capacity of the *R*-group and, as a consequence, they can exhibit metal-specific and cambialistic ‘SOD-activity’ depending on *R*.^6^

Although these complexes are isostructural, their Mn forms are readily distinguished by their Mn(ii) zero-field interactions.^7^ Importantly, this is also true for MnSOD, camSOD and FeSOD with Mn(ii) in their active-sites (herein Mn(Mn)SOD, Mn(cam)SOD and Mn(Fe)SOD).^8^ The zero-field interaction is a magnetic property of paramagnetic species that have more than one unpaired electron and arises from magnetic spin–orbit- and spin–spin interactions of these electrons. It is characterized by its *D* and *E* values and its orientation with respect to the molecular frame. The sum |*D*| + *E* of the three SODs falls into distinct ranges. Mn(Mn)SODs have sums greater than 11.5 GHz, while they are less than 10.9 GHz for the catalytically inactive, Mn(ii) reconstituted FeSODs, (Mn(Fe)SOD).^8^ Mn(cam)SODs and those carrying mutations that alter metal specificity, fall in-between.^8^ Based on the studies of inorganic model systems like those described above, it has been tempting to ascribe these observations to differences in charge polarization. If this is the case, the underlying cause is not discernible at 2.0 Å resolution of crystal structures. The positions of ligand protons relative to the metal centers, in particular those of the water–ligand, should provide valuable information regarding Mn(ii) zero-field interaction of SODs and, more importantly, shed light on their metal specific activity.

**Fig. 2:**
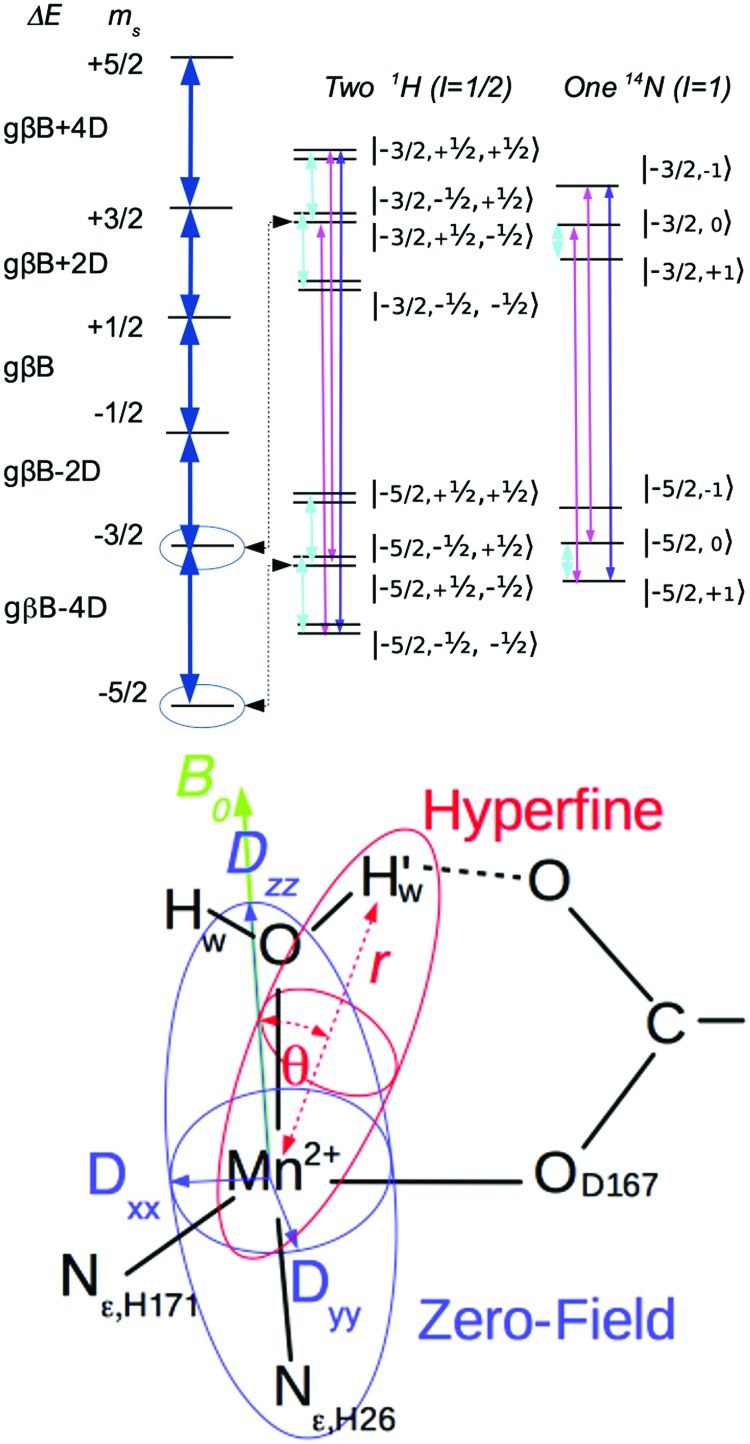
The energy level diagram (top) and geometric arrangement (bottom) of a Mn(ii, *S* = 5/2) coupled to protons (*I* = 1/2) and nitrogens (*I* = 1). On the top, blue arrows correspond to electron spin transitions; the cyan lines, the NMR transitions measured by ENDOR; and magenta and purple lines are those measured by single- and double-quantum ELDOR-NMR. On the bottom, in red, the geometry of the hyperfine interaction between the Mn(ii) and a water ligand proton with the magnetic-field (*B*_0_) applied along the Mn–N_ε,His26_ bond (green) and, in purple, the orientation of the zero-field interaction axes (*D*_*xx*,*yy*,*zz*_) with respect to the Mn(ii) ligands. H81 has been omitted for clarity.

To this end, using high-frequency electron paramagnetic resonance (EPR) techniques we have not only measured the Mn(ii) zero-field interactions of the two active *S. aureus* proteins and the inactive Mn-substituted *E. coli* FeSOD (Mn(Fe)SOD), but also the hyperfine interactions between their Mn(ii) centers and nearby proton and nitrogen nuclear spins. Each hyperfine interaction, *A*_*n*_, is characterized by the size and orientation of its three principal tensor values (*A*_*n*,*mm*_, *m* = *x*, *y*, *z*). These values are the sum of the isotropic (*A*_*n*,iso_) and anisotropic dipolar (*T*_*n,mm*_) contributions. *A*_*n*,iso_ has no explicit dependence on the structure, but is sensitive to the bonding between the metal and the nuclei. By contrast, the dipolar (*T*_*n*_) contribution depends on the distance (*r*_*n*_) between the nucleus and the Mn(ii) electron spin, and the angle (*θ*_*n*_) which their inter-spin vector makes with *B*_0_. As illustrated in Fig. 2, *r*_*n*_ and *θ*_*n*_ are explicitly dependent on the molecular structure. This makes hyperfine interactions an effective structural probe of the metal ligand sphere that can provide potentially finer details than crystallography.^9^ When the nuclei and Mn(ii) are approximated as point-dipoles, as depicted in Fig. 2, the geometric dependence of *A*_*n*_ is easier to appreciate and is given by
1

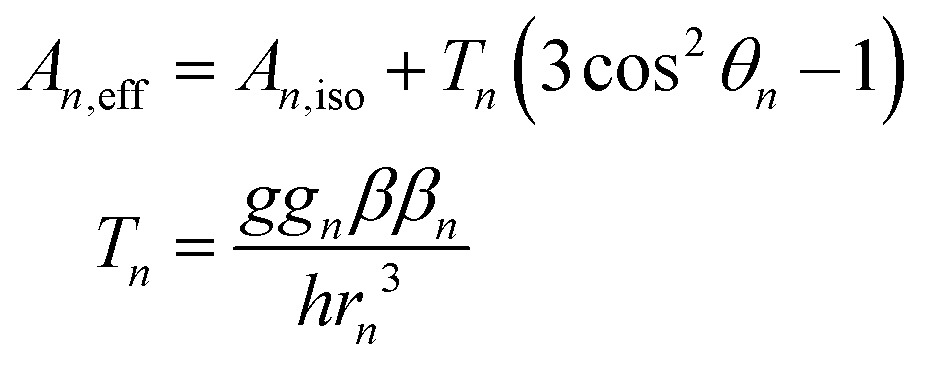




The point-dipole approximation is not completely appropriate because of the proximity of the ^1^H and ^14^N nuclei to the spatially large unpaired electron spin-density of Mn(ii). Even so, eqn (1) does allow for simple estimations and trends. For protons surrounding the Mn(ii) centers in SODs, their Mn(ii) hyperfine couplings are dominated by the dipolar contribution and, hence, determined by their distance to the metal center. Using the SOD crystal structures and assuming standard hydrogen positions on ligands, the largest proton couplings will arise from the two protons on the water ligand with *T*_*n*_ > 3.5 MHz (with *A*_iso_ < 1 MHz), followed by the six imidazole protons (H_2_ and H_5_) that flank the three ligating histidine nitrogens with a *T*_*n*_ of 1.8–2.4 MHz (with *A*_iso_ < 0.1 MHz).^9^–^11^

The hyperfine interactions of the ligand protons and nitrogens are too small to be measured from the EPR spectra directly, but they can be measured using electron nuclear double resonances (ENDOR) and electron double resonance detected NMR (ELDOR-NMR). These techniques, described in detail elsewhere^12^,^13^ and in the ESI†, monitor the change in the EPR signal in response to the excitation of the spins of nuclei surrounding the metal ion, using either the allowed nuclear spin transitions (ENDOR, cyan transition in Fig. 2) or the forbidden simultaneous transition of both the electron and nuclear spins (ELDOR-NMR, magenta and purple transitions). Each nuclear spin will have two resonances at approximately:
2

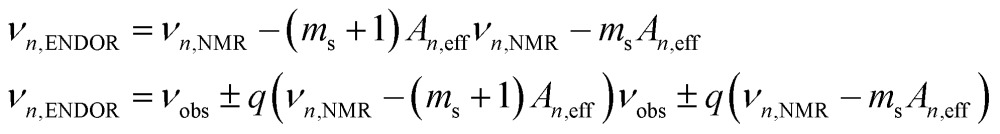




For *S* > 1/2 nuclei such as ^14^N, the nuclear quadrupolar interaction will further split the resonances (see the ESI†). In addition to single quantum (SQ, *q* = 1) transitions, multiple quantum transitions (*q* > 1) can be readily detected by ELDOR-NMR. These not only involve single nuclei, as is the case for nuclei like ^14^N that have a spin greater than 1/2, but also combinations of two or more nuclei, such as two spin 1/2 protons (*q* = 2), and even a DQ transition of a ^14^N and a SQ transition of a ^1^H (*q* = 3). Such multi-nuclei transitions can only arise from nuclei that are coupled to a common Mn(ii), providing structural details not evident in the SQ and ENDOR spectra. One potential problem is that if the *ν*_*n*,NMR_ are small then the SQ and multiple-quantum resonances of different nuclei may overlap. Since *ν*_*n*,NMR_ are proportional to the applied magnetic field, we have exploited sufficiently high magnetic field of 3 T to partially alleviate this problem. In the following, we only consider the positive branch of the ELDOR-NMR spectra, and define *ν*_*n*_′ = *ν*_obs_ + *qν*_*n*,NMR_, which can be subtracted from eqn (2) to obtain *A*_*n*,eff_ in a straightforward manner.

To relate the measured hyperfine couplings to the structure of the active-sites, we have used density functional theory (DFT) calculations on model supramolecular Mn(ii) complexes based on the *S. aureus* crystal structures, as well as many other available SOD crystal structures. This combination of measurements and DFT calculations has allowed us to obtain a detailed picture of the electronic structure of the ligand sphere that suggests that the Mn–H171–E170′ triad may be as important as Q146 in determining the metal specificity of enzymatic activity of Mn- and Fe-dependent SODs.

## Results and discussion

### Crystal structures

**Fig. 3:**
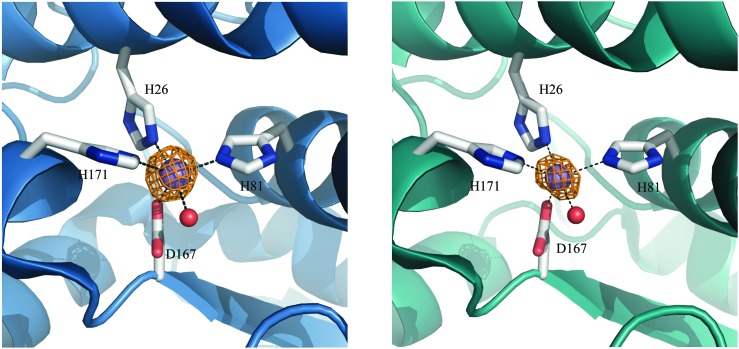
Graphic representation of Mn coordination in the active sites of the homodimers of *S. aureus* (A) MnSOD (ribbon in blue) and (B) camSOD (ribbon in teal). Mn is represented as purple spheres. The orange mesh represents the anomalous difference map rendered at 6.0*σ* and 15.0*σ* on the Mn ions at the active site of MnSOD and camSOD, respectively. Metal-coordinating ligand residues are represented as sticks with carbon atoms colored in grey, oxygens in red, nitrogens in blue and a water molecule represented as a red sphere (PDB accession codes: ; 5N56 for MnSOD and ; 5N57 for camSOD).

Preparations of *S. aureus* MnSOD and camSOD, shown to contain exclusively Mn by ICP-MS, were used in crystallization trials. The crystal structures of Mn(Mn)SOD and Mn(cam)SOD were determined by molecular replacement, using *B. subtilis* SodA as a search model, at 2.07 Å and 2.30 Å, respectively (data collection and refinement statistics are provided in Table S1, ESI†). Two molecules were found in the asymmetric unit of both protein crystals. The dimers of MnSOD and camSOD were superimposable in the protein backbone with an average root mean square deviation (RMSD) of 0.744 Å by least squares fit. Superposition of equivalent Cα atoms of chain A and chain B of MnSOD and camSOD gave positional RMSDs of 0.232 Å and 0.229 Å, respectively. Most of the differences between the structures were found in the loops connecting helical elements within the N-terminal domains. Each monomer presented the characteristic fold conserved amongst Mn/Fe-SODs, with an α-helical N-terminal domain and a C-terminal α/β domain connected by a loop. The active sites were enclosed between the two domains of each monomer, with both domains contributing ligands. As shown in Fig. 3 and as expected, the Mn ions were coordinated in a distorted trigonal bipyramidal geometry by H81, D167, and H171 in the equatorial plane, and H26 and a water molecule in the axial plane (Fig. 3). The bond distances and angles are summarized in Table 1.

**Table 1:** Comparisons of the crystallographic and DFT model structures. DFT-based hyperfine tensors are given in parentheses, in plain-type for ‘as-is’ values and in italics for symmetrized and adjusted values (see text for details)

Model	O_water_–Mn–dihedral (°)			Mn-Interatomic distance (Å)	
	H171	H81	O–C=O (D167)	O_HOH_	O_δ2,D167_
*S. aureus*	30 ± 5	–14 ± 3	19 ± 8	2.22 ± 0.01	2.09 ± 0.03
Mn(Mn)SOD					
*S. aureus*	25 ± 1	–4 ± 2	20 ± 4	2.17 ± 0.04	1.94 ± 0.03
Mn(cam)SOD					
Crystallographic^*a*^	29 ± 8	–15 ± 10	16 ± 10	2.18 ± 0.12	2.01 ± 0.07
				(Mn(Mn)SOD)	
				2.17 ± 0.12	
				(Mn(cam)SOD)	
GO^*b*^	–61	–8	26.3	2.32	2.05
CD^*b*^	33	–8	15	2.30	2.04
CD(NH)^*b*^	29	–8	15	2.32	2.06
CD(OH)^*b*^	29	–8	15	2.33	2.07

Mn-Interatomic distance (Å)					
	N_ε2,H171_	N_ε2,H81_	N_ε2,H26_	H (water ligand)	H (water ligand)
				Free	Hydrogen bound to D167
*S. aureus*	2.31 ± 0.11	2.15 ± 0.02	2.37 ± 0.01		
Mn(Mn)SOD					
*S. aureus*	2.32 ± 0.02	2.33 ± 0.01	2.36 ± 0.01		
Mn(cam)SOD					
Crystallographic^*a*^	2.19 ± 0.07	2.17 ± 0.07	2.20 ± 0.07		
GO^*b*^	2.22	2.18	2.24	2.89	2.61
	^14^N: (1.57, 1.65, 3.33)	^14^N: (1.48, 1.55, 3.29)	^14^N: (2.59, 2.61, 4.40)	(–1.99, –2.44, 7.78)	(–3.71, –4.53, 8.60)
	^1^H_2_: (–2.10, –2.25, 4.39)	^1^H_2_: (–2.07, –2.25, 4.05)	^1^H_2_: (–2.11, –2.29, 4.44)	*(–2.58, –2.58, 7.32)*	*(–3.95, –3.95, 8.35)*
	^1^H_5_: (–1.55, –1.65, 3.73)	^1^H_5_: (–1.78, –1.87, 3.93)	^1^H_5_: (–1.48, –1.57,3.57)		
			*(–1.63, –1.63, 3.71)*		
CD^*b*^	2.20	2.19	2.25	2.97	2.59
CD(NH)^*b*^	2.12	2.21	2.27	2.87	2.63
	^14^N: (0.56, 0.61, 2.31)	^14^N: (1.92, 1.99, 3.73)	^14^N: (2.92, 2.94, 4.74)	(–2.42, –2.92, 7.40)	(–3.70, –4.51, 8.40)
	^1^H_2_: (–1.90, –2.05, 3.87)	^1^H_2_: (–1.98, –2.16, 4.02)	^1^H_2_: (–1.98, –2.14, 4.15)	*(–2.58, –2.58, 7.32)*	*(–3.95, –3.95, 8.35)*
	^1^H_5_: (–1.86, –1.99,4.39)	^1^H_5_: (–1.73, –1.84, 3.89)	^1^H_5_: (–1.57, –1.65, 3.66)		
			*(–1.63, –1.63, 3.71)*		
CD(OH)^*b*^	2.10	2.22	2.27	2.82	2.64
	^14^N: (0.33, 0.35, 2.03)	^14^N: (1.95, 2.03, 3.75)	^14^N: (2.96, 2.98, 4.77)	(–2.75, –3.304, 7.5)	(–3.66, –4.48, 8.30)
	^1^H_2_: (–1.94, –2.10, 3.98)	^1^H_2_: (–1.99, –2.16, 4.03)	^1^H_2_: (–1.95, –2.11, 4.09)		
	^1^H_5_: (–1.86, –2.00, 4.45)	^1^H_5_: (–1.72, –1.82, 3.85)	^1^H_5_: (–1.59, –1.66, 3.68)		

^*a*^Based on average of 57 Mn(Mn)SOD and 4 Mn(cam)SOD structures (see the ESI).

^*b*^See text and Fig. 8 for details.

### EPR spectra

**Fig. 4:**
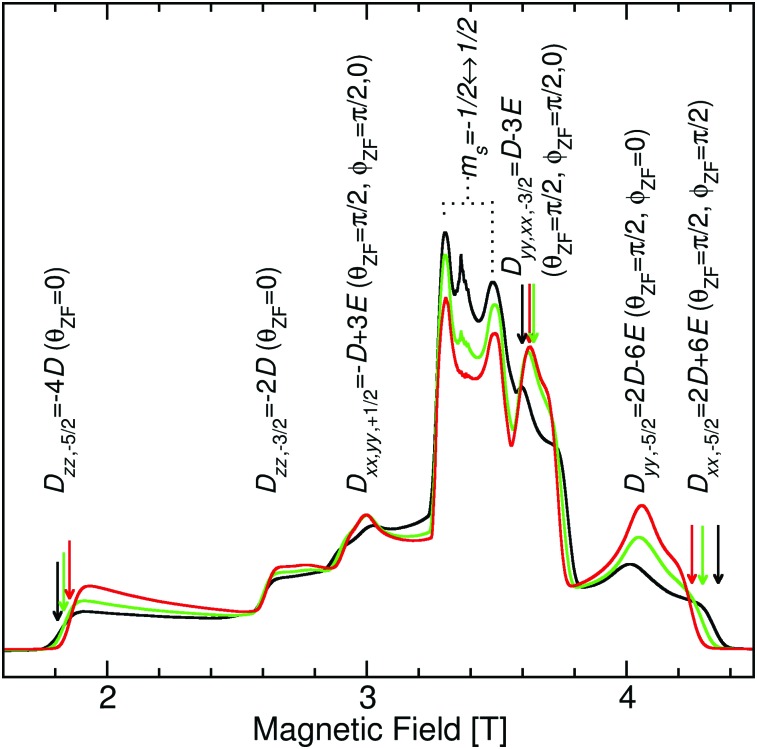
94 GHz 6 K Mn(ii) field-swept echo EPR spectra of: Mn(Mn)SOD (black); Mn(cam)SOD (green); and Mn(Fe)SOD (red). The indicated zero-field field positions are relative to *ν*_obs_/*gβ*. Arrows indicate the magnetic-field positions where the ENDOR and ELDOR-NMR spectra were taken (their colors corresponding to the proteins).

As shown in Fig. 4, *S. aureus* Mn(Mn)SOD and Mn(cam)SOD can be readily distinguished by their 94 GHz field-swept echo EPR spectra. They were also distinct from the spectrum of the inactive Mn(Fe)SOD. This was not completely unexpected based on our previous studies.^8^ The spectra, to first order in the magnetic-field, were essentially determined by their Mn(ii) zero-field with resonant magnetic-field given by
3

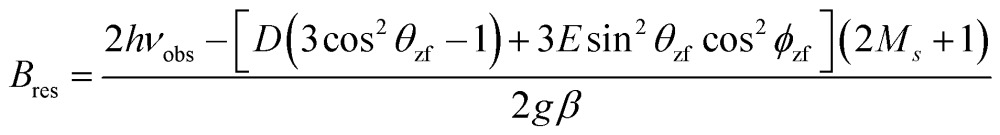

where *ν*_obs_ is the observation frequency (94.0 GHz) and *B*_res_ is the magnetic field at which the *M*_*s*_ ↔ *M*_*s*+1_ (*M*_*s*_ = {–5/2, ±3/2, ±1/2}) electronic spin transitions of the Mn(ii, *S* = 5/2) ion resonate when it is oriented *θ*_zf_ with respect to the zero-field *D*_*zz*_ axis and *φ*_zf_ to *D*_*xx*_ (Fig. 2). What made these spectra unique from our previous work^8^ was that they showed a number of different electronic spin transitions, instead of just *M*_*s*_ = –1/2 ↔ +1/2. This allowed us to essentially ‘read-off’ the zero-field *D* and *E* values using eqn (3) and the field positions labelled *D*_*zz*,–5/2_, *D*_*yy*,–5/2_ and *D*_*xx*,–5/2_ and *D*_*zz*,–3/2_ that originated from the *M*_*s*_ = –5/2 ↔ –3/2 and –3/2 ↔ –1/2 transitions (see the ESI†). For Mn(Mn)SOD, *D* = –10.60 and *E* = 0.63 GHz, similar to *E. coli* Mn(Mn)SOD; for Mn(cam)SOD, *D* = –10.66 and *E* = 0.43 GHz, comparable to the *R. capsulatus* camSOD; and for Mn(Fe)SOD, *D* = –10.46 and *E* = 0.33 GHz, which were the same within errors as those previously obtained from simulations of the *M*_*s*_ = –1/2 ↔ +1/2 transition.^8^

### ENDOR and ELDOR-NMR spectra

Each magnetic-field position on the SOD EPR spectra corresponds to a unique set of orientations of the magnetic-field with respect to the zero-field interaction. The amplitude at a given field position is proportional to the size of the set of orientations. As shown in Fig. 2, the orientations of the zero-field and the hyperfine interactions are fixed to the molecular frame and, hence, to each other. A particular magnetic-field orientation not only fixes *θ*_zf_ and *φ*_zf_, but also *θ*_*n*_ and thereby the size of the hyperfine couplings (*A*_*n*,eff_) (see the ESI† for details) This means that hyperfine measurements at specific points in the MnSOD spectra will be orientation selective^14^—that is the hyperfine interactions are measured in a direction-specific manner. At the extreme edges of the spectra, the set of orientations are single valued: *θ*_zf_ = 0 and *φ*_zf_ = 0 for the low field edge, and *θ*_zf_ = π/2 and *φ*_zf_ = π/2 for the high-field edge. The corresponding *θ*_*n*_ will depend on the relative orientations of the hyperfine and zero-field interaction.

**Fig. 5:**
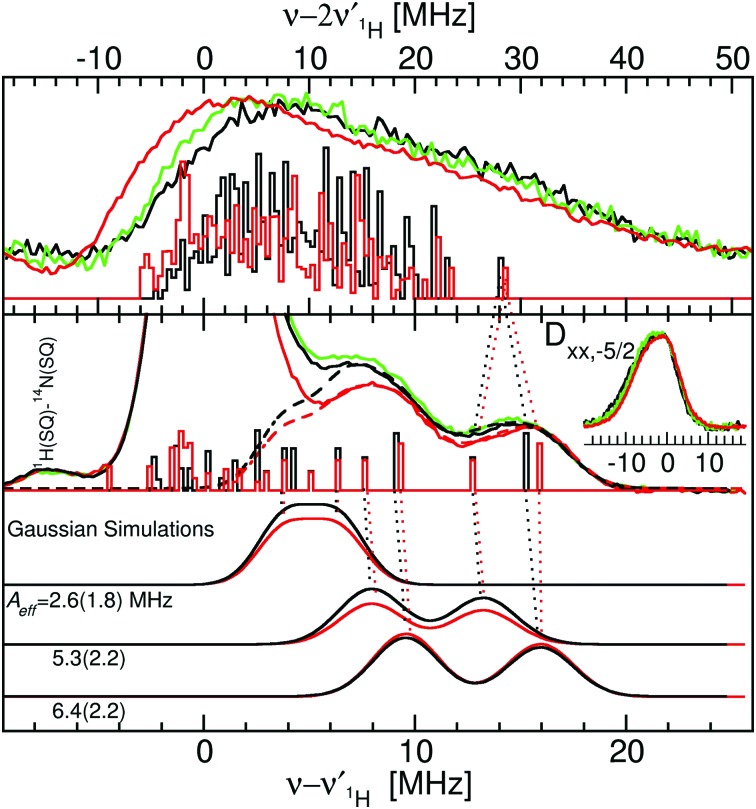
The 94 GHz 5 K SQ (bottom) and DQ (top) ^1^H ELDOR-NMR spectra of Mn(Mn)SOD (black), Mn(cam)SOD (green) and Mn(Fe)SOD (red) taken at the *D*_*zz*,–5/2_ field positions. The SQ spectra taken at the *D*_*xx*,–5/2_ field position are shown in the inset. The exact field positions are indicated by the arrows in Fig. 2. The Gaussian line shape simulations of the SQ spectra are also shown, with their colors corresponding to the measured spectra and their sums as dashed lines. The DFT hyperfine histograms of the GO (black) and CD(NH) (red) models are superimposed and their correspondence to the measured spectra is indicated by the dotted-lines. For the *D*_*zz*,–5/2_ spectra, *ν*_^1^H,NMR_ was 79 MHz and for the *D*_*xx*,–5/2_, it was 185 MHz. See text and the ESI† for details.

**Fig. 6:**
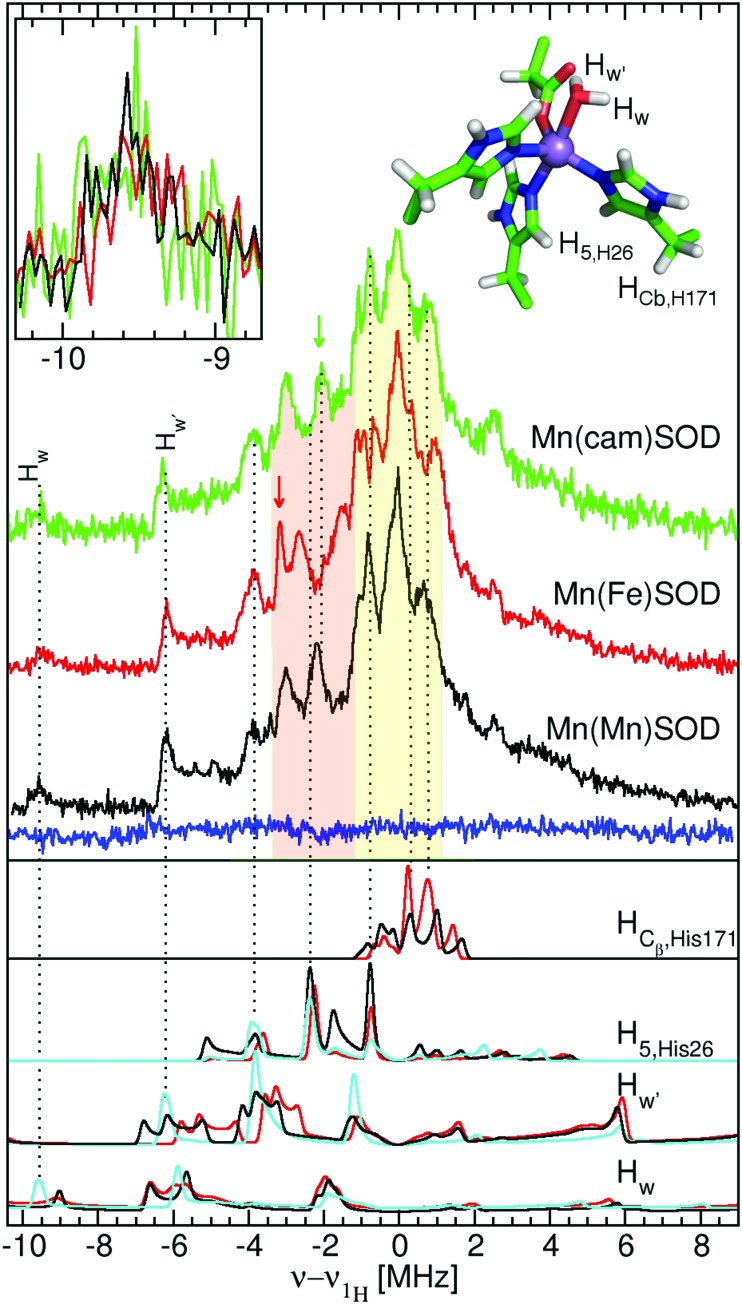
The 94 GHz 5 K Davies ^1^H ENDOR spectra of Mn(Mn)SOD, Mn(Fe)SOD and Mn(cam)SOD taken at the *D*_*yy*,–3/2_ magnet field positions indicated by the arrows in Fig. 2. The blue trace was obtained under the same conditions but at the *D*_–*xx*,–5/2_ magnetic-field position indicated by the black arrow in Fig. 2. The initial electron-spin inversion pulse was 200 ns, followed by a 16 μs radio-frequency pulse and standard spin-echo detection (12 and 24 ns pulses). The lower panel shows the calculated ENDOR spectra based on DFT hyperfine tensors obtained for the GO (red) and CD(NH) (black) models. The cyan traces show the calculated spectra obtained by symmetrizing the DFT hyperfine tensors and manually adjusting the *A*_iso_ values. The calculated spectra have been convolved using 150 kHz Gaussian and each arbitrarily scaled (see text and the ESI† for details).

Fig. 5 shows the ^1^H ELDOR-NMR spectra obtained at these two extremes and Fig. 6, the Davies^12^^1^H ENDOR spectra taken at the *D*_*yy*,–3/2_ magnetic-field position. The corresponding *ν*_^1^H,NMR_ were 79, 185 and 154 MHz. The arrows in Fig. 4 indicate the specific magnetic-fields for each protein where the ELDOR-NMR and ENDOR spectra were obtained. ENDOR spectra at the three fields would have been ideal, since ENDOR resonances are typically sharper than the ELDOR-NMR ones.^11^,^15^ However, the intensity of the ^1^H ENDOR spectra taken at the *D*_*zz*,–5/2_ and *D*_*xx*,–5/2_ field positions was too low to be useful (Fig. 6). This was likely due to our inability to deliver sufficient radio-frequency power to excite the NMR transitions at *ν*_^1^H,NMR_ of 79 and 185 MHz, which were far from 144 MHz for which our system is optimized. By comparison this was not the case for the *D*_*yy*,–5/2_ field position, for which *ν*_^1^H,NMR_ was 154 MHz.

We were struck by the overall similarity of the ^1^H ELDOR-NMR and ENDOR spectra of the three proteins. The *D*_*xx*,–5/2_ spectra (inset of Fig. 5) of Mn(Mn)SOD, Mn(cam)SOD and Mn(Fe)SOD were essentially identical and relatively featureless, extending asymmetrically about *ν*_^1^H,NMR_ from –12.0 to 4.7 MHz, (or an *A*_*n*,eff_ range of –4.8 to 1.8 MHz) for the magnetic-field oriented along the *D*_*xx*_ direction (eqn (3)). In addition to the large matrix signal at *ν* – *ν*_^1^H_ = 0, the *D*_*zz*,–5/2_ spectra had two resolved features at 8 and 16 MHz (Fig. 5; see the ESI† for full spectra). Simulation based on eqn (2) and assuming Gaussian line-shapes showed that these features were unrelated and arose from two different protons with *A*_*n*,eff_ of 5.3 (2.2) and 6.4 (2.2) MHz (with the Gaussian widths in parentheses). The intensity pattern of the *D*_*yy*,–5/2_^1^H ENDOR spectra were also strikingly similar (Fig. 6). All three proteins had nearly identical resonances at –6.3 and –9.2 MHz. The tempting conclusion based on this close similarity of the ^1^H ELDOR-NMR and ENDOR spectra was that the positions of the five ligands at the level of the hydrogen atoms were the same for the three proteins.

This was not the case for Mn(Fe)SOD. As can be seen in Fig. 5, the positive half of all three DQ ^1^H ELDOR-NMR spectra is nearly identical extending to 30 MHz, twice that of the SQ spectra, demonstrating that in all three proteins the resolved high-frequency portion of the SQ spectra arose from two protons from a common Mn(ii) center. The data lend themselves to two possible interpretations. The DQ high-frequency edge was defined by either: (1) two protons having the same *A*_eff_ of 6.4 MHz; or (2) one proton with *A*_eff_ = 5.3 MHz and the other 6.4 MHz. The resolution of the DQ spectra did not allow us to distinguish between these two possibilities. Mn(Fe)SOD also had at least one proton that was distinct. This could be seen in the positions of the low-frequency edges of DQ spectra. The low-frequency edge of Mn(Fe)SOD was 6 MHz lower, indicating that it had at least one pair of protons with a larger combined negative *A*_eff_ than the two active proteins. Both the ^1^H ENDOR and SQ ELDOR-NMR spectra of Mn(Fe)SOD were also distinct in the detail. The simulations of SQ spectra also required a third *A*_*n*,eff_ = 2.6 MHz component to explain the reproducibly lower amplitude of the Mn(Fe)SOD SQ ^1^H ELDOR-NMR spectrum in the 4–8 MHz region. The uniqueness of Mn(Fe)SOD was also evident in the structure of the ENDOR resonances at –3 MHz (orange region, Fig. 6). In the yellow regions, which arise from the smallest *A*_*n*,eff_, all three proteins exhibited subtle but reproducible differences.

**Fig. 7:**
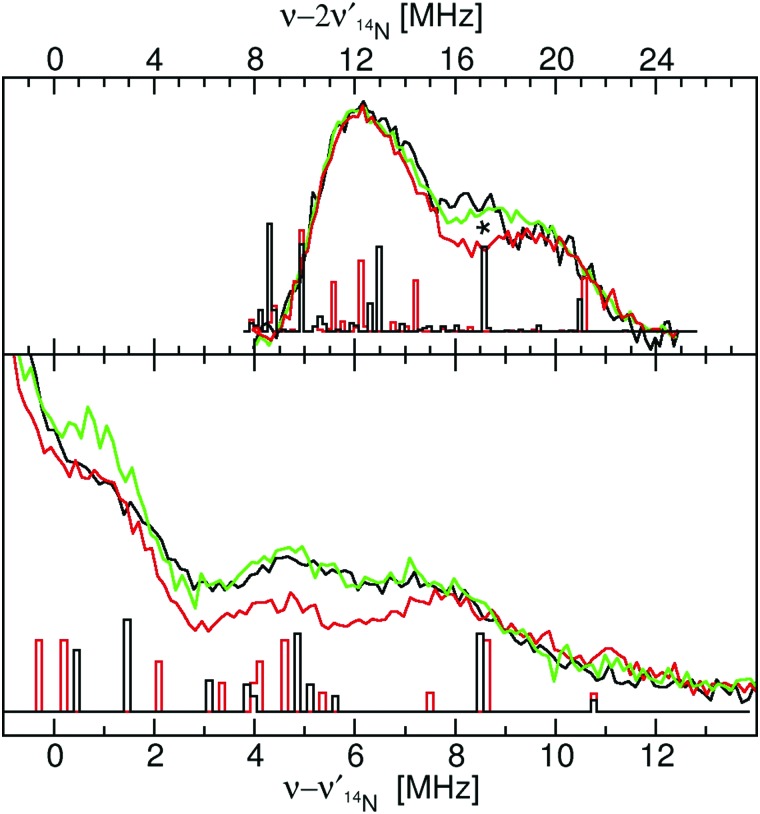
The 94 GHz 5 K SQ (bottom) and DQ (top) ^14^N ELDOR-NMR spectra of Mn(Mn)SOD (black), Mn(cam)SOD (green) and Mn(Fe)SOD (red) taken at the *D*_*zz*,–5/2_ field positions indicated by the arrows in Fig. 2. The DFT hyperfine histograms of the GO (black) and CD(NH) (red) models (*ν*_^14^N,NMR_ = 5.7 MHz).

The close similarity between Mn(Mn)SOD and Mn(cam)SOD and the uniqueness of Mn(Fe)SOD extended to their ^14^N hyperfine interactions (Fig. 7). All three SODs had readily detectable SQ and DQ ^14^N ELDOR-NMR resonances. The large intense center peak (*ν* – *ν*_^14^N_ = 0) obscured the low-frequency portions of SQ resonances, while the low-frequency regions of DQ resonances overlapped with the much larger SQ ones. Nonetheless, it was evident that the three proteins shared a common DQ feature, a partially resolved unequal doublet. The resonance at 20 MHz dictated another at 12 MHz since resonances occur pairwise (eqn (2)). Since, as in the case of radicals,^16^ the 1-spin DQ transition frequencies are purely hyperfine determined and are unaffected by ^14^N nuclear quadrupolar coupling, the doublet corresponded to an *A*_*n*,eff_ of 4 MHz, the largest ^14^N hyperfine coupling when the magnetic-field is along the *D*_*zz*_ direction. The unequal amplitudes of the DQ doublet likely arose from the overlap of the 12 MHz resonance with those arising from smaller *A*_eff_. The three proteins also had a partially resolved SQ doublet with a separation ∼0.5 MHz smaller than 4 MHz. This may have been due to ^14^N nuclear quadrupolar contributions. What made Mn(Fe)SOD stand out was that its SQ and DQ intensity pattern was reproducibly different from those of the active proteins.

### DFT modelling

**Fig. 8:**
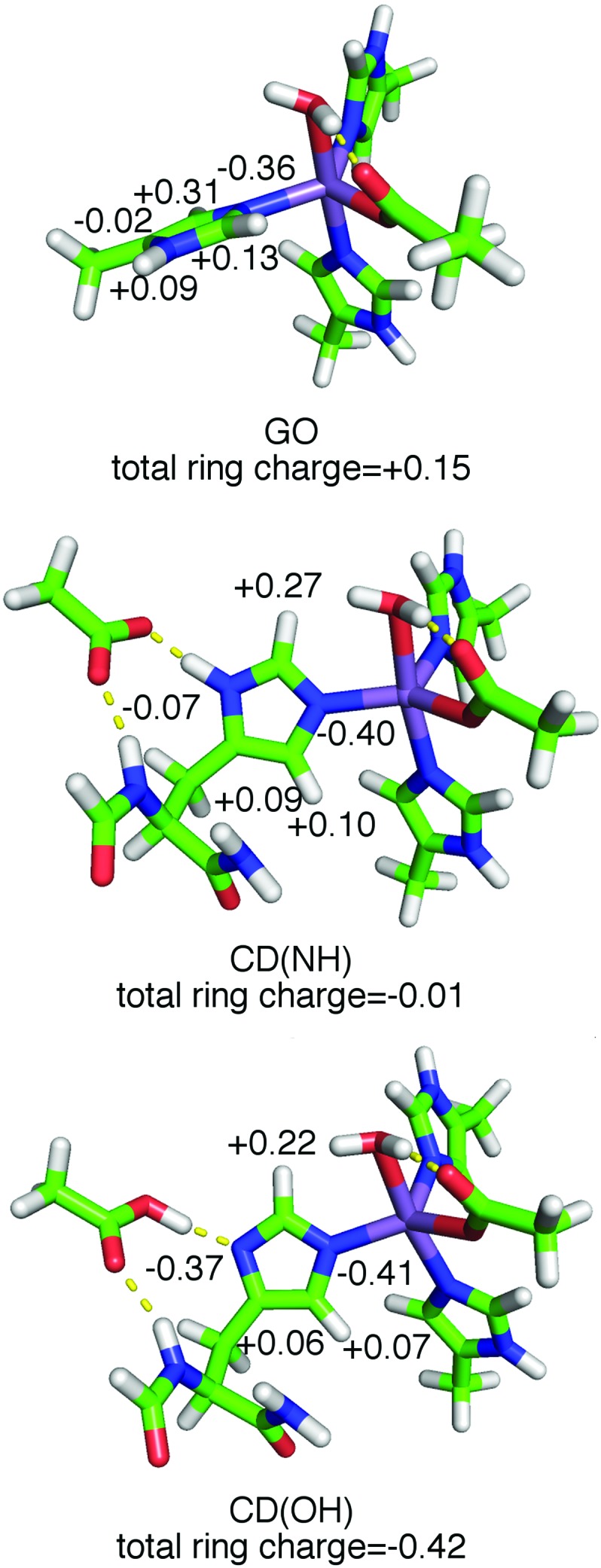
The DFT GO, CD(NH) and CD(OH) model structures. The numbers indicate the CM5 charges of each atom of the His171 ring with their hydrogen charges, if any, summed into them. The orientation of the ligands in Mn(Mn)SOD and Mn(cam)SOD is shown in Fig. 3 and 6.

In order to translate these spectroscopic observations into structural information, the known crystallographic SOD structures and DFT calculations were used to determine the positions of the protons. We also relied on previous ^1^H ENDOR measurements of the concanavalin-A (ConA) Mn(ii) center^9^ for which the water and histidine ligand proton positions were known from neutron diffraction.^17^ The computational models consisted of a Mn(ii) center, with D167 modelled by a propanoate or acetate ligand and the histidines by 4-methylimidazoles (Fig. 1). For reasons that will become apparent, Mn(Fe)SOD was best described by a minimum energy structure (designated GO) which normal mode analysis showed to be a global minimum (Fig. 8). By contrast, Mn(Mn)SOD and Mn(cam)SOD were best described by the model (designated as CD(NH) and CD(OH)) that started from the crystal structure, but energy minimized with the ligand dihedral angles fixed to those found in the crystal structures (see the ESI†). The calculated ligand structures and hydrogen heavy-atom bond-lengths and -angles were entirely consistent with those found in the ConA neutron diffraction structure.^17^ The DFT calculations also returned the theoretical hyperfine and nuclear quadrupolar coupling tensors, in the form of three tensor components, and their orientations with respect to the molecular frame.

However, the measured ENDOR and ELDOR-NMR spectra were obtained relative to the Mn(ii) zero-field interactions of the three proteins and not their molecular frame. Although we knew that the ^1^H ELDOR-NMR spectra in Fig. 5 corresponded to the case where the applied magnetic-field was parallel with the *D*_*zz*_ (*θ*_zf_ = 0), what was not known was how the zero-field interactions were orientated with respect to the molecular frame of the SODs. In simple ionic Mn(ii) complexes, *D*_*zz*_ is often assumed to lie along the axis of greatest symmetry, so we initially placed it along the pseudosymmetry axis, defined by the O_water_–Mn–N_His26_ bonds, and carried out a brute-force search for the Euler angles, the angles of the three rotations that related the zero-field axes to the molecular frame, which yielded the best agreement between the DFT proton hyperfine couplings and ELDOR-NMR and ENDOR spectra. It is assumed that the DFT values were sufficiently accurate to reproduce the measured one. As will be seen, this appeared to the case. The search criteria were: (1) the *A*_^1^H,eff_ values calculated from the DFT reproduce the three couplings found from the simulations of the ELDOR-NMR spectra; and (2) the DFT-based calculated *D*_*yy*,–5/2_ ENDOR spectra match the –5 to –10 MHz region of the measured spectra. These requirements were fulfilled when *D*_*zz*_ was placed along the N_ε,His26_–Mn(ii) bond and the *D*_*xx*_ along the O_δ2,Asp167_–Mn bond as depicted in Fig. 2. The calculated ENDOR spectra based on DFT hyperfine tensors and orientation are shown in Fig. 6. The theoretical results, in the form of histograms that show frequency and transition probability for the magnetic-field oriented along the *D*_*zz*_, are superimposed on the ELDOR-NMR spectra in Fig. 5 and 7 (see the ESI† for methodological details). In both cases, the agreement between the measured and calculated spectra was very good.

The line-shapes of the calculated ENDOR spectra of the water–ligand protons based on the DFT hyperfine tensors were more complex than the measured one. This appeared to be linked to the symmetry of the DFT tensors. Better agreement with the experimental data was obtained by averaging *T*_*xx*_ and *T*_*yy*_—that is by making the dipolar tensor axial (and similar to eqn (1)). This was consistent with single crystal ^1^H ENDOR measurements on the Mn(ii) center in ConA by Carmieli and co-workers.^9^ They showed that the hyperfine tensors of water and histidine ligand protons were symmetric.^9^ For the SODs, small adjustments to the DFT *A*_iso_ were also needed. The biggest was for the Mn(Fe)SOD H_w_ proton (Fig. 6 and Table 1) of 0.4 MHz, while others needed less than 0.1 MHz. This resulted in good agreement between the calculated (cyan traces in Fig. 6) and measured ENDOR spectra. The DFT calculations apparently over-estimated the asymmetry of the hyperfine interactions.

As with the proton hyperfine interactions, the DFT derived ^14^N hyperfine tensors for GO and CD(NH) models accounted for all the salient features of the ^14^N ELDOR-NMR spectra of Mn(Fe)SOD, Mn(Mn)SOD and Mn(cam)SOD. To achieve this agreement required the inclusion of the nuclear quadrupolar coupling obtained from the DFT calculations. The largest hyperfine coupling of 4 MHz was consistent with that of N_ε2,H26_ (Table 1). Interestingly, ^14^N DQ histograms appear to account for the small difference in amplitude at 17 MHz (marked by an asterisk in Fig. 7) between Mn(Fe)SOD and the active proteins. Beyond this, the DFT calculations did not reveal the source of the lower amplitude of the Mn(Fe)SOD ^14^N ELDOR-NMR spectra in the 2–7 MHz region. A more detailed study using HYSCORE spectroscopy is planned to obtain more detailed information regarding Mn–N bonding.

The DFT calculations confirmed that the *A*_eff_ = 6.4 and 5.3 ^1^H ELDOR-NMR components arose from the two water–ligand protons, the larger value from the solvent that forms hydrogen bonds with O_δ1,Asp167_. The ENDOR resonances arising from these protons are respectively labelled as H_w′_ and H_w_ in Fig. 6. The dipolar components of their hyperfine tensors were 4.1 MHz (22°, 2.62 Å) and 3.3 MHz (17°, 2.91 Å), the values in parentheses corresponding to the H–Mn(ii)–O_water_ angles and H–Mn(ii) distances from the DFT calculations. These were close to those measured for concanavalin-A, 3.5 MHz (19°, 2.77 Å) and 4.0 MHz (11°, 3.08 Å), (referred to as H_w1_ and H_w2_, respectively, in Carmieli *et al.*^9^ and the angles and distances coming from a neutron diffraction study^17^).

The calculated ELDOR-NMR transition probabilities were sensitive to geometry, which likely explained the small differences in amplitudes of SQ ^1^H spectra between the active proteins and Mn(Fe)SOD (Fig. 5).

As shown in Fig. 8, the ring plane of H171 in the GO DFT model is tilted –60° relative to the O_water_–Mn–N_His26_ axis compared to +30° in the crystal structures (and, by definition, in the CD(NH) DFT model). This larger dihedral angle causes the angular terms of the dipolar hyperfine couplings (eqn (1)) of H_2_,_His171_ and H_5_,_His171_ to become more negative leading to *A*_eff_ = –1.8 and –0.5 MHz, respectively, in the GO model, compared to –0.9 and +1.6 MHz in the CD(NH) model. It is for this reason that the DQ DFT histogram of the GO model extends to higher negative values than that of the CD models (Fig. 5). This semi-quantitatively matched what was seen in the experimental spectra. Such differences in the ring dihedral angle will manifest themselves most prominently in the orange region of the ^1^H ENDOR spectra in Fig. 6. The calculations showed that the extra feature in the Mn(Fe)SOD marked by the red arrow in Fig. 6 likely arose from the different positions of H_2_,_His171_.

In the same region of the ^1^H ENDOR spectra (Fig. 6), a green arrow marks an instance where none of the three proteins were the same. Based on the DFT calculations, it was tempting to assign this to the angular position of the ring of H26. In the *S. aureus* Mn(Mn)SOD, the ring plane of H26 relative to the Mn–O_δ1,Asp167_ bond was 180°, while in Mn(cam)SOD it was 167°. A prominent resonance in the calculated spectra that coincides with this position arises from H_5_,_His26_. The DFT calculations show that a rotation of H26 about its Mn–N_ε_ bond by 5 to 10° would be sufficient to explain the observed differences. The change in the ^1^H ELDOR-NMR and ENDOR would be on the order of 200 kHz. This would be difficult to discern in the former due to its broad resonances but readily apparent in the latter.

The calculations also showed that any differences in the C_β_ proton hyperfine interactions would contribute to the yellow regions of the ENDOR spectra (Fig. 6). Since these regions were distinct for the three proteins, the positions of these protons, and by extension of the backbones, were unlikely to be the same for Mn(Mn)-, Mn(cam)SODs and Mn(Fe)SOD. This was consistent with the crystal structures of the two *S. aureus* proteins that showed that the positions of C_α_ of the ligands relative to the Mn(ii) were indeed different.

The high level of agreement between the hyperfine measurements, DFT calculations, and the known crystallographic structures allowed us to derive details about the active sites of the three proteins.

### Implications for redox control of activity

The mutation of Q146 to glutamate in *E. coli* FeSOD reverses the hydrogen bonding direction to the water ligand and results in the elongation of the metal-to-water oxygen distance from 2.04 to 2.16 Å in the mutant.^5^ These changes are accompanied by a large increase in *E*^0^ of more than 0.66 V, indicating that Q146 plays an important role in poising the redox potential of the metal center.^5^ Such bond elongation and hydrogen bond reversal would also likely lead to significantly longer Mn(ii)–H_water_ distances, by at least 0.1 Å. The hyperfine measurements show that Mn–H_water_ distances are in fact remarkably constant among Mn(Mn)SOD, Mn(cam) and Mn(Fe)SOD. As can be seen in the inset of Fig. 6, their H_w_ ENDOR resonances are, within the signal-to-noise, centered at the same frequency while the Mn(Fe)SOD H_w′_ resonance is shifted 100 kHz relative to the others. The widths of the H_w_ and H_w′_ resonances were no more than 700 and 400 kHz, respectively. If one assumes that these widths and slightly different centers were solely due to the distribution and differences in Mn(ii)–H distances, the largest expected difference among the three proteins, based on the point dipole approximation, would be substantially less than 0.07 Å (a 100 kHz shift corresponding to 0.03 Å). The water oxygen–Mn(ii) distance is likely to be similarly fixed. This indicates that the interactions between the water–ligand and the protein environment, in particular Q146, must be similar for the active and inactive proteins. Although the Q146–water ligand interaction likely plays an important role in determining the *E*^0^ of the metal centers, we conclude from the hyperfine measurements that they are not the discriminating factor that leads to the different metal-specific activities.

The dihedral-constrained model (CD(NH) and CD(OH), Fig. 8) that we used had a complete H171, with its dihedral angles fixed to crystal structure values and, importantly, the carboxylate sidechain (modelled as an acetate) of E170′, a residue from the other homodimeric subunit. This carboxylate strongly forms hydrogen bonds with both the ring N_*δ*_ of H171 and its backbone amide nitrogen (Fig. 1). Energy minimization of these models yielded two energetically close structures: one with an elongated 1.1 Å N_δ,His165_–H bond (CD(NH)) and the other, favored by only 0.5 kcal mol^–1^, with the proton transferred from the N_*δ*_ to the acetate. Both had a N_δ,His165_–O_acetate_ distance of 2.63 Å indicative of a strong hydrogen bond (CD(OH)). Different attempts to globally optimize models that included E170′ generally led to E170′ disassociating itself from the complex. The hyperfine histograms of CD(NH) and CD(OH) were essentially the same.

The importance of E170 has already been established,^18^ but perhaps underestimated. The *E. coli* MnSOD protein in which E170 is mutated to an alanine contains only iron and is completely inactive even when reconstituted with manganese.^18^ Metal–histidine–carboxylate triads play a well-recognized important role in other metalloenzymes. In the cytochrome *c* peroxidase Fe(ii)–His–Asp triad, a proton is shared by the histidine and aspartate, an arrangement that is readily perturbed by mutations and ionization of nearby residues.^19^ Our data and DFT calculations suggest that in Mn(Mn)- and Mn(cam)SOD, E170′ and H171 share a proton and the negative charge is spread over both, making the H171 imidazole-ring negative. The charge of the ring can range from –0.01 to as much as –0.42 (Fig. 8), based on the CM5 charge model which has been shown to provide useful charge distribution information for systems containing metals.^20^ This Mn–His–Glu triad is likely broken in Mn(Fe)SOD by the rotated position of the H171 ring, as evidenced by the shifted DQ ^1^H spectra. This leads to the negative charge being localized on the E170′ carboxylate sidechain, and gives the H171 ring a charge of +0.15. The altered position of the Mn(Fe)SOD H171 may be related to the subtle differences in the homodimer structure. Charge localization effects of the triad should closely parallel how the electron-donating capacity of the substituent in the [Mn(4′-*R*-terpy)2]^2+^ model affect zero-field interaction and *E*_*m*_. Since in Mn(Mn)SOD and Mn(cam)SOD, E170′ is predicted to have greater electron-donation into H171, these proteins should have a greater zero-field interaction and lower *E*_*m*_ than Mn(Fe)SOD. This is indeed the case for the *E. coli* Mn(Mn)SOD and Mn(Fe)SOD proteins.^2^,^3^,^8^ An appealing physical interpretation of the correlation of |*D*| + *E* with activity also becomes apparent.^8^ Recalling that the zero-field interaction spans –*D* ± 3*E* (eqn (1)) in the equatorial plane of the active-sites, and that *D* < 0, |*D*| + *E* provides a measure of the zero-field interaction that only the direction of the electrostatic influence of the N_ε,His171_/E170′ pair is likely to dominate. The different zero-field interactions for Mn(Mn)SOD and Mn(cam)SOD suggest that there may be even more subtle differences in charge localization in these SODs to which even hyperfine spectroscopy is not sensitive, but that can be readily detected by high-field EPR.

An analysis of the structural data suggests a basis for redox control, and why catalysis by the Mn(Fe)SOD structure may be disrupted. Statistical analysis of the Mn and FeSOD structures (see the ESI†) shows that the metal–O_δ2,E170′_ distances are: 6.97 ± 0.08 Å for MnSODs; 6.93 ± 0.07 Å for Mn and 6.92 ± 0.05 Å for Fe-containing cambialistic SODs; and 6.86 ± 0.07 Å for FeSOD. Although bordering on statistical significance, there is a trend towards longer metal–E170′ distances with Mn. Although the structure of Mn(Fe)SOD is yet to be published, if the tenth of an angstrom difference persists when Fe is replaced by Mn in FeSOD, this may be sufficient to disrupt the E170′–H171 hydrogen bond. Similarly, mutations that seemingly have little to do with redox tuning and that are far from the active site may cause volume changes that modify the E170′–H171 separation, resulting in a shift of catalysis towards ‘Mn’ or ‘Fe’-like behavior. This view provides an alternative or additional cause for the conversion of the *P. gingivalis* cambialistic SOD with high Mn activity to an FeSOD-like one when the G165T (*E. coli* numbering) mutation is introduced over 11 Å away from the active site. This change in specificity has been interpreted as a result of the displacement of W169 by the threonine, which in turn affected the Q146–water ligand interaction.^21^ Comparisons of the Q146–water–metal interactions are complicated by the different metals present in the 1.6 Å resolution structures, Fe in the wild type and Mn in the mutant (PDB ; 1UES and ; 1UER, respectively).^21^ Nonetheless in the crystal structures, although the distances from the metal to H171_Nε_ are the same (2.23 ± 0.06 Å for Mn in the G165T mutant and 2.23 ± 0.03 Å for Fe in the wild-type), the metal to O_δ2,E177′_ distances are different; 7.14 ± 0.09 Å for the cambialistic wild type and 7.02 ± 0.03 Å for the FeSOD-like mutant, following the same trend and exhibiting similar differences as those discussed above: the G165T mutation also shifts the Mn(ii) zero-field interactions from cambialistic to Mn(Fe)SOD values.^22^ Hence, previous structural studies of the *P. gingivalis* camSOD enzyme appear to support our interpretation of our own data, which implicates the importance of the metal–histidine–E170′ triad in determining the metal-specificity of SOD catalysis.

## Conclusions

The active sites of *S. aureus* Mn(Mn)SOD, Mn(cam)SOD and *E. coli* Mn(Fe)SOD can be readily distinguished by their 94 GHz high-field EPR spectra. The 94 GHz ELDOR-NMR and ENDOR of Mn(Mn)SOD and Mn(cam)SOD are remarkably similar, indicating a high-degree of structural homology even at the level of the positions of the protons in proximity to the metal ion, in particular those of the water ligand. The hyperfine spectra of the catalytically inactive Mn(Fe)SOD are different. They show that the ring plane of H171 is at a different angular position compared to the two enzymatically active proteins. This disrupts the alignment of the Mn(ii)–H171–E170′ triad. DFT calculations show that this leads to a different ring charge distribution in comparison to the two active proteins. This shift in charge would be sufficient to change the Mn redox potential of Mn(Fe)SOD to render it inactive.

Application of EPR techniques in combination with DFT calculations has provided us with a physical basis for understanding how the metal specific activities of SODs is determined at the molecular level. The metal specificity of the SODs are not only of fundamental interest, but play a critical role in determining how cells survive oxidative stress, including resisting the mammalian immune system's oxidative burst during infection by pathogens such as *S. aureus*.

## Conflicts of interest

There are no conflicts to declare.

## Supplementary Material

Supplementary information
