# Supplementary material for: A charge polarization model for the metal-specific activity of superoxide dismutases
Source: Phys Chem Chem Phys. 2017 Dec 18;20(4):2363–72. doi: 10.1039/c7cp06829h (PMC5901066; doi:10.1039/c7cp06829h)
Supplement: Supplementary file 1 [file CP-020-C7CP06829H-s001.pdf]

Electronic Supplementary Material (ESI) for Physical Chemistry Chemical Physics.
This journal is © the Owner Societies 2017

A charge polarization model for the metal-specific activity of superoxide dismutases

Anna Barwinska-Sendra, Arnaud Baslé, Kevin J. Waldron, Sun Un

Electronic Supporting Information

1. Sequence alignment of the Mn/Fe-dependent superoxide dismutase enzymes from *S. aureus* and *E. coli.*
2. Crystal parameter table.
3. $D_{zz,-5/2}$ broadband ELDOR-NMR
4. Structural Survey of Mn, Cambialistic and FeSODs and the relationship of DFT structures.
5. Supplementary methods.
6. Coordinates of Geometry Optimized DFT Structures.

1. Sequence alignment of the Mn/Fe-dependent superoxide dismutase enzymes from *S. aureus* and *E. coli*.

```
                      1                  20                  40
S. aureus MnSOD      MAFELPKLPYAFDALEPHFDKETMEIHHDRHHNTYVTKLNAAVEG-TDLESKSIEEIVAN
S. aureus camSOD     MAFKLPNLPYAYDALEPYIDQRTMEFHHDKHHNTYVTKLNATVEG-TELEHQSLADMIAN
E. coli FeSOD (1ISA) -SFELPALPYAKDALAPHISAETIEYHYGKHHQTYVTNLNNLIKG-TAFEGKSLEEIIRS
E. coli MnSOD (1VEW) -SYTLPSLPYAYDALEPHFDKQTMEIHHTKHHQTYVNNANAALESLPEFANLPVEELITK
                      :: ** **** *** *::.  *:* *: .**:***.: *  ::. . :   .: ::: .

                    60                 80                  100
S. aureus MnSOD      LDSVPANIQTAVRNNGGGHLNHSLFWELLSPNS--EEKGTVVEKIKEQWGSLEEFKKEFA
S. aureus camSOD     LDKVPEAMRMSVRNNGGGHFNHSLFWEILSPNS--EEKGGVIDDIKAQWGTLDEFKNEFA
E. coli FeSOD (1ISA) SEG-------GVFNNAAQVWNHTFYWNCLAPNAGGEPTGKVAEAIAASFGSFADFKAQFT
E. coli MnSOD (1VEW) LDQLPADKKTVLRNNAGGHANHSLFWKGLKKGT--TLQGDLKAAIERDFGSVDNFKAEFE
                      :         : **..   **:::*: *  .:     * :   *  .:*:. :** :*

                     120                  140                 160
S. aureus MnSOD      DKAAARFGSGWAWLVVN-NGQLEIVTTPNQDNPLTEGKT------PILGLDVWEHAYYLK
S. aureus camSOD     NKATTLFGSGWTWLVVN-DGKLEIVTTPNQDNPLTEGKT------PILLFDVWEHAYYLK
E. coli FeSOD (1ISA) DAAIKNFGSGWTWLVKNSDGKLAIVSTSNAGTPLTTDAT------PLLTVDVWEHAYYID
E. coli MnSOD (1VEW) KAAASRFGSGWAWLVLK-GDKLAVVSTANQDSPLMGEAISGASGFPIMGLDVWEHAYYLK
                     . *   *****:*** : ..:* :*:*.* ..**           *:: .********:.

                      180                 200
S. aureus MnSOD      YQNKRPDYIGAFWNVVNWEKVDELYNATK
S. aureus camSOD     YQNKRPDYMTAFWNIVNWKKVDELYQAAK
E. coli FeSOD (1ISA) YRNARPGYLEHFWALVNWEFVAKNLAA--
E. coli MnSOD (1VEW) FQNRRPDYIKEFWNVVNWDEAAARFAAKK
                     :.* **.*:  ** :***. .     *
```

Figure S1. Sequence alignment of the Mn/Fe-dependent superoxide dismutase enzymes from *S. aureus* and *E. coli*. Alignment of the amino acid sequences of the three enzymes under study here: the *S. aureus* manganese-specific SOD (MnSOD) (accession number: YP_500165), encoded by the *sodA* gene; the *S. aureus* cambialistic SOD (camSOD) (accession number: YP_498694), encoded by the *sodM* gene; and the *E. coli* iron-dependent SOD (FeSOD) (accession number: AKK12663), encoded by the *sodB* gene, taken from the PDB entry (ID: 1ISA) from the crystal structure[1].The amino acid residues shown to be ligands to the metal cofactor are shown in red, whereas the glutamine (Q146) and glutamate (E170) residues whose importance is described in the main text are highlighted in yellow. Also shown is the sequence of the *E. coli* manganese-specific SOD (MnSOD) (accession number: AKK16012), taken from the PDB entry (ID: 1VEW) from the crystal structure[2], from which the standard numbering system is derived that is used herein. The '*' symbol represents fully conserved sequence positions, whereas the ':' and '.' symbols indicate strongly and weakly conserved sequence positions, respectively. The alignment was generated using the Muscle tool[3]. *S. aureus* (A) MnSOD (PDB 5N56) and (B) camSOD (PDB 5N57 share 74.7 % sequence identity (Figure S1) and are superimposable with a core RMSD of 0.746 Å (data not shown).

2. Table S1. Data collection and refinement statistics for *S. aureus* Mn(Mn)SOD and Mn(cam)SOD.

**Data collection**	MnSOD	camSOD
Date collected	24/04/14	23/02/14
Source	I04	I04
Wavelength (Å)	1.881	1.282
Space group	$P2_1$	$P6_1$
Cell dimensions		
a, b, c (Å)	51.3, 68.4, 56.7	142.2, 142.2, 46.4
α, β, γ (°)	90.0, 99.4, 90.0	90.0, 90.0, 120.0
No. of measured reflections	77487 (1527)	78873 (7817)
No. of independent reflections	21845 (936)	24136 (2339)
Resolution (Å)	43.30-2.07 (2.12-2.07)	46.55-2.30 (2.38-2.30)
$CC_{1/2}$	0.996 (0.953)	0.968 (0.760)
$I/\sigma I$	9.0 (1.5)	7.0 (1.5)
Completeness (%)	91.6 (51.7)	99.8 (100.0)
Redundancy	3.5 (1.6)	3.3 (3.3)
**Refinement**		
R_{work}/ R_{free}	15.0 (23.9)	19.2 (23.7)
No. atoms		
Protein	3202	3242
Ligand/Ions	2	2
Water	140	121
B-factors		
Protein	39.3	43.9
Ligand/Ions	27.3	30.1
Water	38.6	35.2
R.m.s deviations		
Bond lengths (Å)	0.0143	0.0169
Bond angles (º)	1.6	1.7
PDB code	5N56	5N57

Values in parentheses are for the high-resolution shell.

3. $D_{zz,-5/2}$ broadband ELDOR-NMR

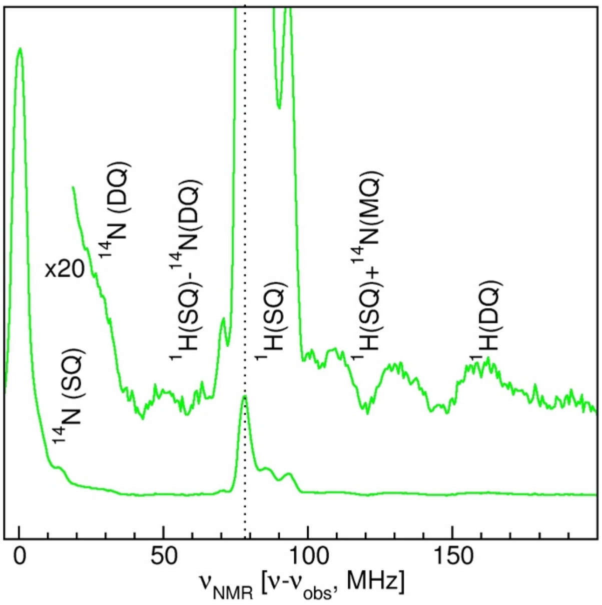

Figure S2. The 5 K 94 GHz Mn(II) ELDOR-NMR spectrum of Mn(cam)SOD obtained at the $D_{zz,-5/2}$ magnetic-field position of the EPR spectrum. The inset shows the ^{1}H ELDOR-NMR spectrum taken at the $D_{xx,-5/2}$ field position. Dotted black line indicates $\nu_{^1H}$.

4. Structural survey of Mn, cambialistic and FeSODs used for entries in Table 1.

Sixty-four structures of the Mn, cambialistic and FeSODs found in the PDB database were examined. Each structural element, calculated from the structures, were assumed to be statistically independent. PDB accession codes used in the analysis of the crystallographic data. Many of the structures were of proteins carrying a mutation. However, the maximum standard deviations, ±10º for dihedral angles, ±0.12 Å for the Mn(II)-Water bond-lengths and ±0.07Å for the other bond-lengths, were only modestly larger than the variation seen among the wild-type *S. aureus*, *E. coli* and *H. sapiens* MnSODs. The averages and standard deviations given in Table 1 show a conservative estimate of the variation in the structural parameters.

Mn containing structures:
1ap5, 1ap6, 1ar4, 1em1, 1en4, 1en5, 1en6, 1gn4, 1gv3, 1i08, 1i0h, 1ix9, 1ixb, 1ja8, 1kkc, 1luv, 1luw, 1n0j, 1n0n, 1pl4, 1pm9, 1qnm, 1szx, 1ues, 1var, 1vew, 1xdc, 1xil, 1xre, 1zsp,

1zte, 1zuq, 2a03, 2adp, 2adq, 2aw9, 2cdy, 2ce4, 2p4k, 2qka, 2qkc, 2rcv, 3ak2, 3bfr, 3c3s, 3c3t, 3dc5, 3dc6, 3kky, 3lsu, 3mds, 3qvn, 4br6, 4c7u, 4e4e, 4f6e, 4gun, 4jzg, 4x9q, 5a9g.

Cambialistic enzyme structures with Mn
1ar4, 1ues, 3ak2, 4jzg

Cambialistic enzyme structures with Fe
1ar5, 1bs3, 1bt8, 1uer, 4yio, 1avm, 1bsm, 1qnn, 3ak3, 4yip.

Fe containing structures
1b06, 1coj, 1gn6, 1ids, 1isa, 1isb, 1ma1, 1my6, 1unf, 1wb7, 1wb8, 1y67, 1za5, 2bkb, 2bpi, 2cw2, 2cw3, 2goj, 2gpc, 2nyb, 2w7w, 3cei, 3esf, 3h1s, 3js4, 3lio, 3ljf, 3rn4, 3tjt, 3tqj, 4dvh, 4f2n, 4ffk, 4h3e, 4jyy, 4l2a, 4l2b, 4l2c, 4l2d, 4yet.

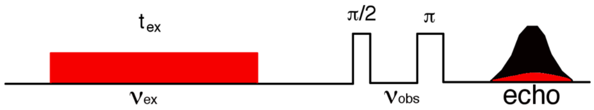

Figure S3. The ELDOR-NMR experiment. A pulse with frequency ν_{ex} and duration t_{ex} excites a forbidden transition inverting the populations of the two states. The excitation of the forbidden transition reduces the intensity of the electron spin echo (from black to red) detected at ν_{obs}.

5. Supplementary Methods

EPR Spectroscopy.

The 94 GHz EPR and ELDOR-NMR spectra were obtained at 5 K using a Bruker Elexsys II 680 EPR spectrometer equipped with a "power upgrade 2" and an Oxford Instruments CF935 flow cryostat. The EPR spectra were obtained by measuring the amplitude of a standard two-pulse Hahn echo ($t_p(\pi)$=24 ns and interpulse time of 400 ns) as a function of the magnetic-field.

The ELDOR-NMR spectra were obtained by measuring the integrated two-pulse echo intensity detected at ν_{obs} as a function ν_{ex} (Figure S3). They were typically taken overnight. Microwave cavity tuning, and the lengths and powers of the excitation and detection pulses

are listed below. The choices in these parameters were dictated by efficiency and spectra resolution. Spectra were typically taken overnight.

Table S3. Acquisition Conditions

	Cavity Tuning Frequency[1]	t_{ex} (μs)[2]	π_{ex} (ns)[3]	π_{obs} (ns)[4]
^{14}N SQ, D_{zz}	ν_{obs}	20	1600	240
^{14}N SQ, D_{xx}	ν_{obs}	6	160	200
^{14}N DQ, D_{zz}	ν_{obs}	2	200	200
^{1}H SQ, D_{zz}	$\nu_{obs}+\nu_{NMR}$	2	210	200
^{1}H SQ, D_{xx}	$\nu_{obs}+½\nu_{NMR}$	2	200	200
^{1}H DQ, D_{zz}	$\nu_{obs}+\nu_{NMR}$	2	70	200
Broad	$\nu_{obs}+\nu_{1H,NMR}$	2	-	200

1. The cavity was over-coupled to approximately the same extent for all samples.
2. Length of excitation pulse.
3. π-pulse time measured at the $\nu_{obs}+\nu_{NMR}$ for SQ and $\nu_{Detect}+2\nu_{NMR}$ for DQ spectra.
4. π-pulse time at observation frequency (ν_{obs}).

The normalization methods of the ELDOR-NMR are also listed in Table S3. For SQ ^{14}N, it was sufficient to use the center transition which took into account number of scans, detection gain and sample concentration. For the ^{1}H SQ, the center transition was too far to use for normalization. Normalization accounting for number of scans, sample concentration, excitation power and detection gain resulted in about 10% variation from sample to sample and among the three proteins. It was found that the best basis for comparison was to simply normalize the spectra to the height of the ^{1}H matrix. The ^{1}H and ^{14}N DQ spectra were sensitive to excitation power. This was exacerbated by the need to over-couple the cavity, which was difficult to do in a reproducible way, and the variation in the microwave power over the width of the DQ resonances. For ^{1}H DQ, nutation measurements were taken at $2\nu'_{1H}$, $2\nu'_{1H} + 15\ MHz$ and $2\nu'_{1H} + 30\ MHz$ to ensure the excitation powers and their variation over the width of the DQ resonance were as similar as possible for the three proteins. Although the shapes of the resonance could be reproduced, there were appreciable sample to sample variations in the overall amplitude of the signals. To compensate for this

variation, the ^{1}H DQ spectra were normalized to their integrated amplitude. For ^{14}N DQ, it was evident that the shape of the Mn(Fe)SOD spectrum was different.

All ENDOR spectra were recorded using the method of Davies.[4] They were obtained with a 200-ns microwave preparation pulse followed by a 16-μs radiofrequency pulse and two-pulse spin-echo detection (12- and 24-ns microwave pulses separated by 400 ns) and with a shoot repetition time of 11 ms. Spectra typically took overnight.

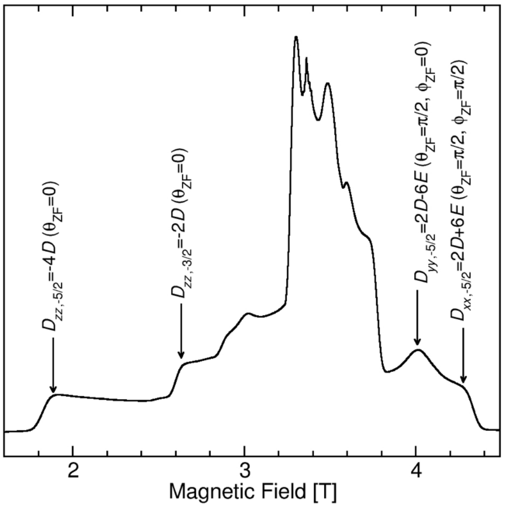

Figure S4. Field positions used to calculate the Mn(II) *D* and *E* values.

Determination of D and E values from spectra. The zero-field *D* and *E* values were obtained directly from the spectra using the three $D_{nn,-5/2}$ (*nn=xx, yy* and *zz*) field positions shown in Figure S4. These positions are given with respect to ν/gβ (where ν is the microwave frequency, g=2.0010[5] and β=13.996246 GHz/T). The values could be 'read-off' using the first order equations:

$$D^1_{xx,m} = \frac{2\nu - (6m_s + 3)E - (2m_s + 1)D}{2g\beta}$$

$$D^1_{yy,m} = \frac{2\nu + (6m_s + 3)E - (2m_s + 1)D}{2g\beta}$$

$$D^{1}_{zz,m} = \frac{\nu + (2m_s + 1)D}{g\beta}$$

However, since |D|/ν was significantly large, the equations for the field positions to second-order in *D* and *E* were required to achieve greater accuracy. They were derived from the work of Bir[6] and are:

$$D^{2}_{xx,m} = \frac{16\nu^2 - (12{m_s}^2 + 12m_s - 29)E^2}{16\nu g\beta} - \frac{(24\,\nu(2m_s + 1) - 2(12{m_s}^2 + 12m_s - 29)D)E}{16\nu g\beta} - \frac{(12{m_s}^2 + 12m_s - 29)D^2 - 8\nu(2m_s + 1)D}{16\nu g\beta}$$

$$D^{2}_{yy,m} = \frac{16\nu^2 - (12{m_s}^2 + 12m_s - 29)E^2}{16\nu g\beta} - \frac{(-24\,\nu(2m_s + 1) + 2(12{m_s}^2 + 12m_s - 29)D)E}{16\nu g\beta} - \frac{(12{m_s}^2 + 12m_s - 29)D^2 - 8\nu(2m_s + 1)D}{16\nu g\beta}$$

$$D^{2}_{zz,m} = \frac{4\nu^2 - (12{m_s}^2 + 12m_s - 29)E^2 + 4\nu(2m_s + 1)D}{4\nu g\beta}$$

.

The $D_{zz,-3/2}$ field position was also useful. First, the first-order estimate of *E* was obtained from the difference of the $D_{xx,-5/2}$ and $D_{yy,-5/2}$ field positions. This *E* value was used to estimate *D* to second-order using the difference, $D_{zz,-3/2}$- $D_{zz,-5/2}$, and the corresponding second-order equations. The second-order *D* value was then used to obtain a second-order estimate of *E*. The last two steps were repeated after which the values became self-consistent. The use of differences rather than absolute field positions removed systematic errors arising the measurement of the magnetic-field.

Analytical expressions for the orientations contributing to the $D_{-3/2,yy}$ magnetic-field position. The orientations of the magnetic-field with respect to the zero-field interaction that

contribute to the $D_{-3/2,yy}$ magnetic-field position can be derived from Eq.1. For the m_s=-5/2↔-3/2 transition, they are:

$$\frac{E}{D} = \frac{6cos^2\theta - 1}{6cos(2\varphi)sin^2\theta + 3}$$

the m_s=-3/2↔-1/2 transition:

$$\frac{E}{D} = \frac{cos^2\theta}{cos(2\varphi)sin^2\theta + 1}$$

and m_s=+1/2↔+3/2 transition:

$$\frac{E}{D} = \frac{3cos^2\theta - 2}{3cos(2\varphi)sin^2\theta - 3}$$

For the ENDOR calculations, the second-order versions of the expressions were used. These did not lend themselves to simple analytical solution like the three above and required numerical solutions.

DFT Calculations. Density functional theory (DFT) calculations were carried out using Gaussian 09 (Revision B.01)[7] and ORCA[8]. The former was used to optimize geometries using the B3LYP[9–12] /6-31+G(D,P) [13–16] hybrid density functional and basis-set and default options. The latter was used to obtain the hyperfine coupling values using the PBE0[17]/6-31+G(D,P)[13–16] combination and the tightSCF and Grid5 options. Normal mode analysis showed that the GO model was a true minimum energy structure. CM5 charges were calculated using Gaussian.

Calculation of ENDOR and ELDOR-NMR spectra. The hyperfine tensors and their orientations obtained from the DFT calculations were used to calculate the ENDOR and ELDOR-NMR spectra. They were used to solved the spin Hamiltonian:

$$H = \omega_e S_z + D_0 \left[3S_z - S(S+1)\right] + D_{+1}\left[S_z S_+ + S_+ S_z\right] + D_{-1}\left[S_z S_- + S_- S_z\right] +$$

[illegible]

The D_N are functions of D, E, θ_{zf} and ϕ_{zf} and are given in reference [6]. The zero-field D and E values were those obtained from field-swept spin echo spectra described above. For the ^{14}N

$$H_Q = \frac{e^2qQ}{4I(2I-1)}(3I_z^2 - I^2)\left[\frac{1}{2}(3\cos^2\theta - 1) + \eta\sin^2\theta\cos 2\phi\right]$$

nuclei, the quadrupolar interaction term

were also included. The DFT–derived orientations of the quadrupolar interactions for the three ligating nitrogens were the same for all model structures, approximately: one axis along the Mn(II)-N bond, another perpendicular to the ring-plane and the third in the mutually perpendicular direction (in the ring plane and perpendicular to the Mn(II)-N bond). This approximate orientation was used rather than those obtained from the DFT calculations to simplify the calculation.

For each orientation of the magnetic-field (one for $D_{zz,-5/2}$ ELDOR-NMR and 2000 for $D_{yy,-3/2}$ ENDOR simulations) the resulting matrix forms of the spin Hamiltonian (12x12 for protons, and 18x18 for ^{14}N) were numerically solved for their eigen-vectors and -values using LAPCK95 interfaced to locally written Fortran and Python programs. The echo-detected Davis ^{1}H ENDOR spectra were simulated as previously described.[18] The transition probabilities were calculated as products of the nuclear (Δm_I =1) and electronic (Δm_S =1) spin transition probabilities. For the ELDOR-NMR, all possible transition energies and probabilities between the two relevant m_s-manifolds were calculated. For example, for a transition between $\left|-\frac{5}{2}, l\right\rangle \leftrightarrow \left|-\frac{3}{2}, l\right\rangle$ "detection" transition, we consider $\left|-\frac{5}{2}, l \pm 1\right\rangle \leftrightarrow \left|-\frac{3}{2}, l\right\rangle$, $\left|-\frac{5}{2}, l\right\rangle \leftrightarrow \left|-\frac{3}{2}, l \pm 1\right\rangle$, $\left|-\frac{5}{2}, l \pm 2\right\rangle \leftrightarrow \left|-\frac{3}{2}, l\right\rangle$ and $\left|-\frac{5}{2}, l\right\rangle \leftrightarrow \left|-\frac{3}{2}, l \pm 2\right\rangle$ "pump" transitions (Fig 2). The latter two corresponded to 1-spin DQ transitions. The energies of these four forbidden transitions relative to energy of the allowed transition corresponded to the ELDOR-NMR resonance frequency. For each proton, this amounted to two resonances above and below the allowed transition, and for each ^{14}N nuclei, because of the quadrupolar

coupling, four SQ resonances above and below the corresponding allowed transition. The energies of the 1-spin ^{14}N DQ were independent of quadrupolar coupling. For simplicity, the ELDOR intensities were assumed to be the product of detect and pump transition probabilities calculated from the eigenfunctions. The energies of the two-nuclei double quantum resonances were assumed to be the sums of the two corresponding single quantum transitions and the product of their transition probabilities was taken to be the intensity of this type of double quantum transitions. The resonance energies and transition amplitude were used to generate the ELDOR-NMR histograms. For clarity, the ^{14}N histograms only contain contributions for ligating nitrogens and the ^{1}H histograms the water and imidazole protons.

Cloning, expression and purification of the SOD enzymes.

The cloning of the *S. aureus sodA* gene (ORF SAOUHSC_01653), encoding MnSOD, and the *sodM* gene (ORF SAOUHSC_00093), encoding camSOD, has been described previously. (Garcia et al., 2017).

The *Escherichia coli sodB* gene, encoding FeSOD, was amplified from *Escherichia coli* K12 MG1655 genomic DNA using Phusion DNA polymerase (NEB) with the primer pair introducing 5' *Nco*I and 3' *Xho*I restriction sites (Table S4). The genetic insert was cloned into *Nco*I/*Xho*I (NEB) digested pET29a *E. coli* expression vector (Novagen). After sequencing (GATC Biotech, Germany), site-directed mutagenesis with sodB-qc1_F and sodB-qc1_R primer pair was used to correct for an introduced Gly2 residue (insertion of G5, C6 bases) and revert to the wild type sequence, which was confirmed by sequencing.

Expression and purification of the *S. aureus* SODs was essentially as described (Garcia et al., 2017) with minor modifications. *E. coli* BL21 (λDE3) cells (Table S5) transformed with pET29a-sodA and pET29a-sodM constructs were cultured at 37ºC with 180 rpm orbital shaking in lysogeny broth (LB) medium containing 5 mM $MnCl_2$ and protein expression was

induced by addition of 1 mM isopropyl β–D-1-thiogalactopyranoside (IPTG) followed by incubation for 4 h under the same conditions. After purification as described (Garcia et al., 2017), in order to yield a sample of protein that contained exclusively manganese, proteins were unfolded in 2.5 M guanidine hydrochloride, 5 mM EDTA, 20 mM 8-hydroxyquinoline pH 3.8 [1], to remove bound metal ions, followed by refolding through several rounds of dialysis against 20 mM Tris, 100 mM NaCl, 10 mM $MnCl_2$, pH 7.5, as described (Garcia et al., 2017). Aliquots of each purified protein were resolved on an analytical Superdex S200 Increase 10/30 column (GE Healthcare) in 20 mM Tris, 150 mM NaCl, 5 mM EDTA, pH 7.5. Protein concentrations of eluant fractions were determined from A_{280nm} measurements, using extinction coefficients (ε_{280nm}) of 62,681 M^{-1} cm^{-1} for SodA and 64,949 M^{-1} cm^{-1} for SodM, which were empirically-determined from quantitative amino acid analysis (Alta Bioscience).

E. coli FeSOD protein was expressed in an *E. coli* BL21 (λDE3) Δ*sodA* strain, which was created with P1 phage-mediated transduction[19] of the (*sodA*::MudPR13)25 allele from PN134 strain background[20], kindly provided by Dr James A. Imlay, Illinois. The pET29a-sodB construct was transformed into *E. coli* BL21 (λDE3) Δ*sodA* cells and selected on LB agar plates containing 50 μg ml^{-1} kanamycin, 10 μg ml^{-1} chloramphenicol (*sodA*::cam). For protein expression, cells were cultured at 37ºC with 180 rpm orbital shaking in M9 minimal medium[21] containing 0.2% Casamino acids, 0.25 mM $MnCl_2$ and selective antibiotics (50 μg ml^{-1} kanamycin, 10 μg ml^{-1} chloramphenicol). At OD_{600nm} ~0.5, protein expression was induced by addition of 1 mM IPTG and 0.25 mM $MnCl_2$ followed by incubation for 4 h under the same conditions. Cells were harvested by centrifugation (20 min, 4,000 g, 4ºC), washed in 20 mM Tris, pH 7.5, 10 mM EDTA, followed by a further wash in 20 mM Tris, pH 7.5, 150 mM NaCl and stored at -20ºC.

Cells were resuspended in 20 mM Tris, pH 7.5 and lysed by sonication (6 x 10 s cycles at 14-micron amplitude, with 1 min intervals on ice) and the lysate was clarified by

centrifugation (20 min, 19,000 *g*, 4ºC). The soluble lysate was initially resolved by anion exchange chromatography (AEC) eluted with 0-1 M NaCl linear gradient elution on a 5 mL HiTrap Q HP column (GE Healthcare). Collected fractions were analyzed for protein by SDS-PAGE. Fractions containing FeSOD were pooled and further purified with a second round of AEC in 20 mM 2-(N-morpholino)ethanesulfonic acid (MES) pH 6.5 buffer on a 5 mL HiTrap Q HP column with a linear 0-1 M NaCl gradient elution. The peak AEC fractions containing the recombinant protein were subsequently resolved by size exclusion chromatography (SEC) on a preparative Superdex S200 16/60 column, in 20mM MES, 150 mM NaCl, pH 6.5 buffer.

Protein concentrations of eluant fractions were determined from A_{280nm} measurements, using the theoretical extinction coefficient (ε_{280nm}) for FeSOD of 51,910 M^{-1} cm^{-1} (ExPasy, Protparam tool).

Table S4. Oligonucleotides' sequences

Primer name	5'-3' sequence
sodB-amp-NcoI_F	GCGTCCATGGgcTCATTCGAATTACC
sodB-amp-XhoI_R	GCGTCTCGAG TTA TGC AGC GAG
sodB-qc1_F	GCC ACG CGG TTC CAT GTC ATT CGA ATT ACC TG
sodB-qc1_R	CAG GTA ATT CGA ATG ACA TGG AAC CGC GTG GC

Table S5. ***E. coli* strains used through this study**

Strain	Genotype
**DH5α**	*E. coli fhuA2 lac(del)U169 phoA glnV44 Φ80' lacZ(del)M15 gyrA96 recA1 relA1 endA1 thi-1 hsdR17*
**BL21(λDE3)**	*E. coli B dcm ompT hsdS*($r_B^- m_B^-$) *gal*
**BL21(λDE3) Δ*sodA***	*E. coli* BL21(λDE3) (*sodA*::MudPR13)25

Elemental analysis of recombinant proteins by inductively coupled plasma mass-spectrometry (ICP-MS)

Analysis of the metal content of purified proteins was performed by ICP-MS using a Thermo x-series instrument operating in collision cell mode as previously described (Garcia et al., 2017). Stoichiometry of metal to protein was determined by comparing quantified analytes with the protein concentration (A_{280nm}).

SOD activity assay

SOD activity was assessed qualitatively *in-gel* by nitrotetrazolium blue chloride negative staining of purified protein samples resolved on non-denaturing 12% polyacrylamide gels[6] as previously described (Garcia et al., 2017).

Preparation of protein samples for EPR

The purified protein samples for EPR experiments were treated with 10 mM EDTA at room temperature for 30 minutes to remove free manganese contamination. EDTA was removed by buffer exchange through centrifugal ultrafiltration (Amicon Ultra-15, 10 kDa MWCO) before the final concentration step to ~ 2 mM with VivaSpin 500 (10 kDa MWCO) concentrators. To reduce the oxidation state of protein-bound manganese, samples were treated with a fresh solution of sodium dithionite (Sigma). MnSodA was treated with 10-molar equivalents of dithionite and MnSodM with 2-molar equivalents of dithionite. Reduced protein samples were flash-frozen in liquid nitrogen and stored at -80°C.

Crystallization of *S. aureus* MnSOD and camSOD

Mn-loaded preparations of recombinant MnSOD (SodA) and camSOD (SodM) were subjected to crystallization screening using a Mosquito liquid handling robot (TTP Labtech) with commercially available matrix screens: PACT, JCSG+ and Structure (Molecular Dimensions) in 96-well MRC crystallization plates (Molecular Dimensions), using the sitting-drop of vapor-diffusion method, incubated at 20°C.

The initial conditions producing crystals were subsequently optimized for salt, polyethylene glycol (PEG) precipitant concentration and pH, using the hanging drop of vapor diffusion method, incubated at 20°C, in 24-well Linbro plates (Molecular Dimensions). Drops

were set up manually at 1 μl + 1 μl and at 2 μl + 1 μl of protein and crystallization liquor, respectively, per test condition, each equilibrated against 500 μl of reservoir solution. Diffracting crystals were obtained in 200 mM $MgCl_2$, 30% (w/v) PEG 4000 and 100 mM Tris pH 8.5 for Mn(Mn)SOD and in 50 mM potassium thiocyanate and 34% (w/v) PEG 2000 MME for Mn(cam)SOD. Samples were cryo-protected using the reservoir solution complemented with 20% PEG 400. X-ray diffraction data were collected at the Diamond Light Source synchrotron (Didcot, UK). The data were processed using XDS[22] and scaled using Aimless (Evans, 2013). The phase problem was solved by molecular replacement using Molrep[23], using the search model 2RCV. The pdb models were completed by iterative cycle of refinement in Refmac[24] in tandem with manual model rebuilding in COOT[25]. The PDB codes for Mn(Mn)SOD and Mn(cam)SOD are 5N56 and 5N57, respectively. The data collection and refinement statistics are summarized in Table S1. All crystallographic images were generated using PyMOL Molecular Graphics System, Version 1.8 (Schrödinger, LLC).

References

[1] M. S. Lah, M. M. Dixon, K. A. Pattridge, W. C. Stallings, J. A. Fee, M. L. Ludwig, *Biochemistry* **1995**, *34*, 1646–1660.
[2] R. A. Edwards, H. M. Baker, M. M. Whittaker, J. W. Whittaker, G. B. Jameson, E. N. Baker, *J. Biol. Inorg. Chem.* **1998**, *3*, 161–171.
[3] R. C. Edgar, *Nucleic Acids Res.* **2004**, *32*, 1792–7.
[4] E. R. Davies, *Phys. Lett. A* **1974**, *47*, 1–2.
[5] S. Un, L. C. Tabares, N. Cortez, B. Y. Hiraoka, F. Yamakura, *J. Am. Chem. Soc.* **2004**, *126*, 2720–2726.
[6] G. L. Bir, *Sov. Physics-Solid State* **1964**, *5*, 1628–1635.
[7] M. J. Frisch, G. W. Trucks, H. B. Schlegel, G. E. Scuseria, M. A. Robb, J. R. Cheeseman, G. Scalmani, V. Barone, B. Mennucci, G. A. Petersson, et al., **2010**.
[8] F. Neese, *Wiley Interdiscip. Rev. Comput. Mol. Sci.* **2012**, *2*, 73–78.
[9] A. D. Becke, *Phys. Rev. A* **1988**, *38*, 3098–3100.
[10] C. Lee, W. Yang, R. G. Parr, *Phys. Rev. B* **1988**, *37*, 785–789.
[11] A. D. Becke, *J. Chem. Phys.* **1993**, *98*, 5648.
[12] A. D. Becke, *J. Chem. Phys.* **1993**, *98*, 1372.
[13] R. Ditchfield, *J. Chem. Phys.* **1971**, *54*, 724.
[14] W. J. Hehre, R. Ditchfield, J. A. Pople, *J. Chem. Phys.* **1972**, *56*, 2257.
[15] V. A. Rassolov, J. A. Pople, M. A. Ratner, T. L. Windus, *J. Chem. Phys.* **1998**, *109*,

1223.
[16] P. C. Hariharan, J. A. Pople, *Theor. Chim. Acta* **1973**, *28*, 213–222.
[17] C. Adamo, V. Barone, *J. Chem. Phys.* **1999**, *110*, 6158.
[18] A. Potapov, D. Goldfarb, *Inorg. Chem.* **2008**, *47*, 10491–10498.
[19] J. H. Miller, *Experiments in Molecular Genetics*, Cold Spring Harbor Laboratory, **1972**.
[20] J. A. Imlay, S. Linn, *J. Bacteriol.* **1987**, *169*, 2967–76.
[21] J. Sambrook, D. W. (David W. Russell, *Molecular Cloning : A Laboratory Manual*, Cold Spring Harbor Laboratory Press, **2001**.
[22] W. Kabsch, *Acta Crystallogr. Sect. D Biol. Crystallogr.* **2010**, *66*, 125–132.
[23] A. Vagin, A. Teplyakov, *Acta Crystallogr. Sect. D Biol. Crystallogr.* **2000**, *56*, 1622–1624.
[24] M. D. Winn, G. N. Murshudov, M. Z. Papiz, *Methods Enzymol.* **2003**, *374*, 300–321.
[25] P. Emsley, B. Lohkamp, W. G. Scott, K. Cowtan, *Acta Crystallogr. Sect. D Biol. Crystallogr.* **2010**, *66*, 486–501.

6. Geometry Optimized Structure. Listed below are coordinates of the four structures obtained using B3LYP/6-31+G(D,P).

GO:

Mn	0.266514	-0.202493	-0.259219
O	0.927115	0.485507	-2.369820
O	0.281975	1.765765	0.305965
C	0.118882	2.832073	-0.409510
O	-0.011856	2.816501	-1.656687
N	2.115298	-1.355957	-0.252279
N	-0.336985	-0.796385	1.816831
N	-1.683705	-0.649911	-1.184569
C	3.066050	-1.254313	-1.172766
C	-0.391416	0.114309	2.777649
C	-2.410035	0.307414	-1.747523
N	-3.598389	-0.191804	-2.165817
C	-3.651517	-1.543455	-1.861050
C	-2.449865	-1.803312	-1.250716
N	-0.814712	-0.448615	3.936387
C	-1.050701	-1.797944	3.720297
C	-0.745985	-1.988559	2.395340
N	4.126169	-2.026157	-0.836466
C	3.857662	-2.663093	0.366397
C	2.602756	-2.229429	0.710161
C	-4.823265	-2.409779	-2.189074
C	4.820694	-3.590917	1.031747
C	-1.535116	-2.724249	4.787366
C	0.089773	4.147422	0.355914
C	-0.110518	5.393842	-0.505971
H	0.479170	0.118537	-3.147968
H	0.599881	1.438772	-2.273856
H	-0.698061	4.063906	1.117540
H	1.031142	4.206710	0.919395
H	2.026026	-2.478701	1.588498
H	4.979724	-2.112725	-1.374046
H	3.011690	-0.636040	-2.056558
H	-2.091514	-2.746203	-0.862877
H	-4.327072	0.340341	-2.625297
H	-2.106773	1.339562	-1.868062
H	-0.802769	-2.906962	1.827628
H	-0.938418	0.040084	4.814335
H	-0.140406	1.158337	2.655597
H	-1.641853	-3.734746	4.383564
H	-0.835935	-2.774594	5.631254
H	-2.512860	-2.417099	5.178818
H	-4.635067	-3.433039	-1.853168
H	-5.738279	-2.059492	-1.695732
H	-5.014153	-2.443346	-3.268783
H	4.390185	-3.968399	1.963173
H	5.056454	-4.455391	0.398845

H	5.763413	-3.087977	1.280194
H	-0.108077	6.292396	0.121000
H	0.685458	5.492708	-1.250425
H	-1.062014	5.356406	-1.046125

CD(minimal):

Mn	0.000711	0.058512	-0.326849
O	0.673322	0.862384	-2.379082
N	-0.581708	-0.566439	1.758578
N	-1.890164	-0.690822	-1.171526
N	1.826930	-1.142051	-0.376486
O	0.281521	1.945386	0.400827
C	0.342822	3.086003	-0.212764
O	0.162710	3.219138	-1.443508
C	-1.054833	-1.756778	2.290949
C	-0.449858	0.266856	2.780544
N	-0.817656	-0.342951	3.934643
C	-1.211666	-1.642755	3.650166
C	-2.145411	-1.178794	-2.376894
N	-3.477358	-1.368517	-2.538842
C	-4.128209	-0.977198	-1.379027
C	-3.121144	-0.561264	-0.544960
C	-5.611308	-1.038997	-1.213511
C	-1.684267	-2.600610	4.694667
C	0.623715	4.297445	0.664779
C	0.680505	5.631487	-0.079480
C	2.645108	-1.253645	-1.414364
N	3.674180	-2.081036	-1.112525
C	3.519776	-2.534941	0.189217
C	2.362509	-1.940253	0.623821
C	4.484789	-3.464024	0.849992
H	0.210075	0.720337	-3.219401
H	0.493242	1.842757	-2.145997
H	-0.148101	4.316907	1.446852
H	1.567448	4.099646	1.191741
H	1.884877	-2.028919	1.588344
H	4.441460	-2.311080	-1.731599
H	2.537228	-0.728677	-2.351798
H	-3.200812	-0.173839	0.460023
H	-3.922235	-1.735171	-3.371275
H	-1.415563	-1.419689	-3.136544
H	-1.257126	-2.615749	1.666447
H	-0.810946	0.085798	4.851814
H	-0.100916	1.286593	2.702635
H	-1.941274	-3.557713	4.232715
H	-0.913008	-2.791735	5.451155
H	-2.577601	-2.229890	5.212503
H	-5.888201	-0.685599	-0.216754
H	-6.127337	-0.406525	-1.946184
H	-5.990764	-2.062347	-1.323034
H	4.150541	-3.684746	1.867317

H	4.567205	-4.416415	0.311810
H	5.488646	-3.026728	0.917103
H	0.885175	6.448517	0.621451
H	1.467036	5.627115	-0.840507
H	-0.265889	5.842686	-0.586906

CD(NH)

O	1.326245	1.792908	2.074179
Mn	1.869584	-0.022708	0.728376
O	2.493395	-0.987598	2.440857
C	2.301791	-0.732025	3.692286
O	1.586761	0.200089	4.123029
N	3.422132	1.325338	-0.093275
C	3.444852	2.636875	0.084705
N	2.630565	-1.829879	-0.407600
C	3.323360	-2.795704	0.173246
N	0.031686	0.107391	-0.335798
C	-0.718487	1.194195	-0.575519
N	-1.840181	0.885854	-1.236915
C	-1.855112	-0.479143	-1.427234
C	-2.929132	-1.172699	-2.217846
C	-3.844078	-2.158622	-1.444867
N	-4.639057	-1.432696	-0.461238
C	-2.986298	-3.309236	-0.879617
N	-2.704749	-3.291383	0.453027
N	4.424095	3.197344	-0.668530
C	5.070173	2.194907	-1.377768
C	4.426673	1.042210	-1.004234
N	3.607513	-3.776270	-0.722277
C	3.064805	-3.427568	-1.951682
C	2.468506	-2.211773	-1.729874
C	-0.684566	-0.948667	-0.872657
C	6.204576	2.458025	-2.313334
C	2.986501	-1.667874	4.673734
C	3.160157	-4.294893	-3.163898
O	-2.493938	-4.132685	-1.649311
C	-5.620229	-2.034641	0.257720
O	-5.834901	-3.245326	0.294944
O	-3.653778	2.654098	-1.718097
C	-4.793725	2.409851	-1.186999
C	-5.816506	3.542091	-1.255790
O	-5.118280	1.331195	-0.629885
H	2.783618	-1.365656	5.702818
H	0.398377	2.073979	2.016488
H	1.380986	1.284904	2.954279
H	2.629688	-2.692494	4.516610
H	4.068011	-1.667178	4.495180
H	4.616613	0.030611	-1.331886
H	4.645171	4.184064	-0.701133
H	2.798266	3.182724	0.755922
H	-0.324652	-1.964861	-0.816034

H	-2.649490	1.605689	-1.476466
H	-0.457578	2.208121	-0.305397
H	1.915055	-1.601046	-2.428967
H	4.102795	-4.634073	-0.516419
H	3.603126	-2.811430	1.216581
H	2.671349	-3.802852	-4.009199
H	4.202060	-4.492314	-3.447070
H	2.661445	-5.260413	-3.011657
H	-2.467773	-1.750814	-3.025658
H	-3.566440	-0.415350	-2.686503
H	6.546703	1.517431	-2.754032
H	5.910003	3.122654	-3.135455
H	7.060128	2.915611	-1.800323
H	-6.757439	3.250931	-0.782672
H	-5.414951	4.434296	-0.760870
H	-5.998237	3.810887	-2.303071
H	-4.504158	-2.641550	-2.176081
H	-4.672349	-0.400249	-0.516917
H	-6.216170	-1.310524	0.840862
H	-2.305569	-4.140283	0.833948
H	-3.300734	-2.756739	1.070057

CD(OH)

O	-1.493954	-2.225797	1.997027
Mn	-1.800069	-0.308387	0.700202
O	-2.547320	0.603460	2.404064
C	-2.499010	0.273586	3.650261
O	-1.882857	-0.718726	4.100549
N	-3.369478	-1.512869	-0.310658
C	-3.487397	-2.825525	-0.195246
N	-2.371633	1.597607	-0.395711
C	-3.064645	2.572061	0.168624
N	0.100844	-0.517303	-0.172524
C	0.816497	-1.645339	-0.399435
N	2.019077	-1.421213	-0.932581
C	2.117241	-0.049230	-1.050627
C	3.301225	0.610324	-1.704220
C	4.199660	1.509342	-0.813648
N	4.859668	0.698768	0.206258
C	3.378044	2.699254	-0.278584
N	2.978813	2.658627	1.020606
N	-4.433559	-3.290808	-1.050197
C	-4.954686	-2.220020	-1.762678
C	-4.274562	-1.128122	-1.285486
N	-3.192226	3.618296	-0.688907
C	-2.539756	3.303631	-1.873621
C	-2.043298	2.041157	-1.666253
C	0.936355	0.500322	-0.591153
C	-6.020395	-2.369696	-2.798707
C	-3.238013	1.193081	4.609577
C	-2.450716	4.241558	-3.032546

O	3.031721	3.590892	-1.051794
C	5.808472	1.189697	1.043942
O	6.139979	2.369257	1.129919
O	3.751104	-3.395109	-1.147494
C	4.869040	-3.239024	-0.460215
C	5.756677	-4.464908	-0.488538
O	5.175688	-2.212902	0.145133
H	-3.115653	0.856352	5.640826
H	-0.560165	-2.492773	1.977454
H	-1.607485	-1.762107	2.892746
H	-2.859865	2.216949	4.509130
H	-4.304384	1.211819	4.354936
H	-4.372246	-0.093787	-1.581330
H	-4.711388	-4.258719	-1.145894
H	-2.930673	-3.438350	0.498560
H	0.644137	1.538597	-0.524495
H	3.118782	-2.561964	-1.078667
H	0.437097	-2.641492	-0.202391
H	-1.450034	1.435611	-2.336658
H	-3.655543	4.493640	-0.483127
H	-3.453908	2.547953	1.176129
H	-1.896737	3.768205	-3.847834
H	-3.441621	4.517639	-3.415889
H	-1.920698	5.165223	-2.767407
H	2.963534	1.248203	-2.529000
H	3.936107	-0.166515	-2.145661
H	-6.268049	-1.390664	-3.218614
H	-5.698325	-3.014303	-3.626635
H	-6.941900	-2.794569	-2.380244
H	6.677005	-4.275512	0.066366
H	5.226569	-5.317412	-0.049027
H	5.993009	-4.730104	-1.524973
H	4.958707	1.970873	-1.456709
H	4.754865	-0.318472	0.171952
H	6.262289	0.400550	1.670705
H	2.586443	3.508892	1.403733
H	3.432035	2.026684	1.665049
